# Antibody responses and correlates of protection in the general population after two doses of the ChAdOx1 or BNT162b2 vaccines

**DOI:** 10.1038/s41591-022-01721-6

**Published:** 2022-02-14

**Authors:** Jia Wei, Koen B. Pouwels, Nicole Stoesser, Philippa C. Matthews, Ian Diamond, Ruth Studley, Emma Rourke, Duncan Cook, John I. Bell, John N. Newton, Jeremy Farrar, Alison Howarth, Brian D. Marsden, Sarah Hoosdally, E. Yvonne Jones, David I. Stuart, Derrick W. Crook, Tim E. A. Peto, A. Sarah Walker, David W. Eyre, the COVID-19 Infection Survey team, Tina Thomas, Daniel Ayoubkhani, Russell Black, Antonio Felton, Megan Crees, Joel Jones, Lina Lloyd, Esther Sutherland, Emma Pritchard, Karina-Doris Vihta, George Doherty, James Kavanagh, Kevin K. Chau, Stephanie B. Hatch, Daniel Ebner, Lucas Martins Ferreira, Thomas Christott, Wanwisa Dejnirattisai, Juthathip Mongkolsapaya, Sarah Cameron, Phoebe Tamblin-Hopper, Magda Wolna, Rachael Brown, Richard Cornall, Gavin Screaton, Katrina Lythgoe, David Bonsall, Tanya Golubchik, Helen Fryer, Stuart Cox, Kevin Paddon, Tim James, Thomas House, Julie Robotham, Paul Birrell, Helena Jordan, Tim Sheppard, Graham Athey, Dan Moody, Leigh Curry, Pamela Brereton, Ian Jarvis, Anna Godsmark, George Morris, Bobby Mallick, Phil Eeles, Jodie Hay, Harper VanSteenhouse, Jessica Lee, Sean White, Tim Evans, Lisa Bloemberg, Katie Allison, Anouska Pandya, Sophie Davis, David I. Conway, Margaret MacLeod, Chris Cunningham

**Affiliations:** 1 Nuffield Department of Medicine, University of Oxford, Oxford, UK; 2 Big Data Institute, Nuffield Department of Population Health, University of Oxford, Oxford, UK; 3 The National Institute for Health Research Health Protection Research Unit in Healthcare Associated Infections and Antimicrobial Resistance at the University of Oxford, Oxford, UK; 4 Health Economics Research Centre, Nuffield Department of Population Health, University of Oxford, Oxford, UK; 5 The National Institute for Health Research Oxford Biomedical Research Centre, University of Oxford, Oxford, UK; 6 Department of Infectious Diseases and Microbiology, Oxford University Hospitals NHS Foundation Trust, John Radcliffe Hospital, Oxford, UK; 7 Office for National Statistics, Newport, UK; 8 Office of the Regius Professor of Medicine, University of Oxford, Oxford, UK; 9 Health Improvement Directorate, Public Health England, London, UK; 10 Wellcome Trust, London, UK; 11 Nuffield Department of Orthopaedics, Rheumatology and Musculoskeletal Sciences, University of Oxford, Oxford, UK; 12 MRC Clinical Trials Unit at UCL, University College London, London, UK; 13 Oxford University Hospitals NHS Foundation Trust, Oxford, UK; 14 University of Manchester, Manchester, UK; 15 IQVIA, London, UK; 16 National Biocentre, Milton Keynes, UK; 17 Glasgow Lighthouse Laboratory, London, UK; 18 Department of Health and Social Care, London, UK; 19 Welsh Government, Cardiff, UK; 20 Scottish Government, Edinburgh, UK; 21 Public Health Scotland, Edinburgh, UK

**Keywords:** Viral infection, Epidemiology, Antibodies, SARS-CoV-2, Vaccines

## Abstract

Antibody responses are an important part of immunity after Coronavirus Disease 2019 (COVID-19) vaccination. However, antibody trajectories and the associated duration of protection after a second vaccine dose remain unclear. In this study, we investigated anti-spike IgG antibody responses and correlates of protection after second doses of ChAdOx1 or BNT162b2 vaccines for severe acute respiratory syndrome coronavirus 2 (SARS-CoV-2) in the United Kingdom general population. In 222,493 individuals, we found significant boosting of anti-spike IgG by the second doses of both vaccines in all ages and using different dosing intervals, including the 3-week interval for BNT162b2. After second vaccination, BNT162b2 generated higher peak levels than ChAdOX1. Older individuals and males had lower peak levels with BNT162b2 but not ChAdOx1, whereas declines were similar across ages and sexes with ChAdOX1 or BNT162b2. Prior infection significantly increased antibody peak level and half-life with both vaccines. Anti-spike IgG levels were associated with protection from infection after vaccination and, to an even greater degree, after prior infection. At least 67% protection against infection was estimated to last for 2–3 months after two ChAdOx1 doses, for 5–8 months after two BNT162b2 doses in those without prior infection and for 1–2 years for those unvaccinated after natural infection. A third booster dose might be needed, prioritized to ChAdOx1 recipients and those more clinically vulnerable.

## Abstract (web summary)

A large study in the United Kingdom shows that virus-specific antibody levels associated with at least 67% protection against SARS-CoV-2 Delta variant infection last longer after two doses of BNT162b2 vaccine than after two doses of ChAdOx1 vaccine in previously uninfected individuals.

## Main

The Pfizer-BioNTech BNT162b2 and Oxford-AstraZeneca ChAdOx1 nCoV-19 (hereafter ChAdOx1) SARS-CoV-2 vaccines have been widely used in the United Kingdom (UK) and worldwide^1,2^. In the UK, vaccines were initially prioritized to older adults, frontline health and social care workers, and clinically vulnerable individuals, and then offered to other adults in decreasing age order^3^. Up to 4 October 2021, 85% and 78% of the population (aged ≥12 years) have received one and two doses, respectively^4^.

With widespread Alpha variant transmission, in January 2021 the UK government extended the dosing interval from 3–4 weeks to 12 weeks for all vaccines to maximize first dose coverage, based on preliminary data showing high short-term efficacy from single BNT162b2 (90%) and ChAdOx1 (70%) doses^5^. This approach raises several questions. Although the ChAdOx1 trial found higher vaccine efficacy with dosing intervals of at least 6 weeks^6^, BNT162b2 trials did not compare different dosing intervals. Subsequent UK studies showed that extended BNT162b2 dosing intervals generated higher antibody responses than the 3-week interval^7–9^. However, these studies were based on relatively small sample sizes (*n* < 600) or specific population groups such as healthcare workers, potentially reducing generalizability, and antibody levels were measured only at specific times after second doses. With the rapid emergence of the Delta variant, and greater protection after second than first vaccine doses^10–12^, from mid-2021 dosing intervals were reduced to 8 weeks^13^ to achieve greater protection faster. However, large population-based investigations of how these different dosing intervals, or other factors, affect longer-term antibody changes after the second dose are limited, but they are essential to assess the duration of protection and the need for booster doses.

After the ChAdOx1 vaccine, anti-spike IgG and pseudovirus neutralization titers are associated with protection against symptomatic SARS-CoV-2 infection^14^. Similarly for the mRNA-1273 vaccine, anti-spike antibody and neutralization titers were inversely associated with SARS-CoV-2 infection, with 68.5% of vaccine efficacy up to 126 days after the second dose mediated by day 29 neutralization titers^15^. However, how these measurements relate to antibody levels and durations of protection in populations over time, and after BNT162b2 vaccination, is not fully understood.

We used data from the UK’s national COVID-19 Infection Survey (ISRCTN21086382), a large representative sample of households with longitudinal follow-up, to investigate longer-term anti-trimeric spike IgG antibody responses after second ChAdOx1 or BNT162b2 vaccinations, and we quantified the effect of dosing interval, age and prior infection status on antibody peak levels and declines. We estimated the association between anti-spike IgG levels and protection against SARS-CoV-2 infection and combined these findings with estimated antibody trajectories to predict the duration of protection after second vaccination and natural infection.

## Results

**Extended Data Fig. 1:**
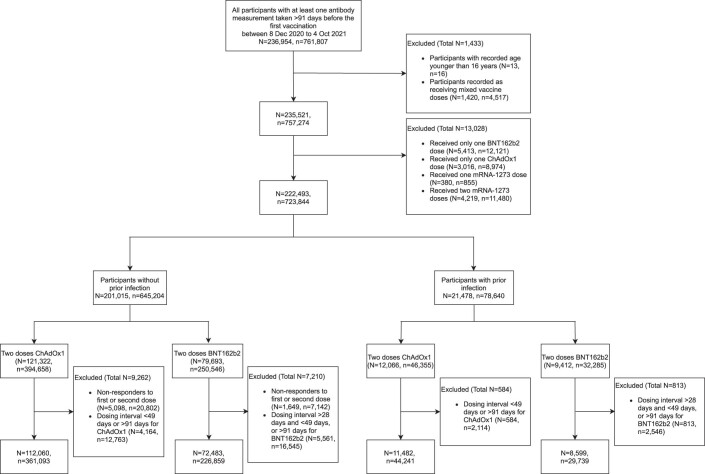
Flowchart of the study cohort. *N* represents the number of participants, and *n* represents the number of antibody measurements.

From 8 December 2020 to 4 October 2021, 222,493 participants received two ChAdOx1 or two BNT162b2 vaccinations and had at least one antibody measurement from 91 days before the first vaccination onward. The median (interquartile range (IQR)) age was 57 (43–68) years; 120,866 (54.3%) were female; 209,898 (94.3%) reported white ethnicity; 7,071 (3.2%) reported working in patient-facing healthcare; and 62,814 (28.2%) reported having a long-term health condition. In total, 121,322 (54.5%) and 79,693 (35.8%) participants without evidence of prior infection (Methods) received two doses of ChAdOx1 or BNT162b2, as did 12,066 (5.4%) and 9,412 (4.2%) participants with evidence of prior infection, respectively. These four cohorts contributed 723,844 anti-spike IgG measurements (Extended Data Fig. 1). The median (IQR) [range] dosing interval was 76 (68–78) [17–237] and 76 (66–78) [17–225] days for those receiving ChAdOx1 without or with prior infection, respectively, and 71 (58–77) [17–289] and 65 (56–76) [17–238] days, respectively, for BNT162b2 (Supplementary Table 1).

### Anti-spike IgG response after first and second dose

**Extended Data Fig. 2:**
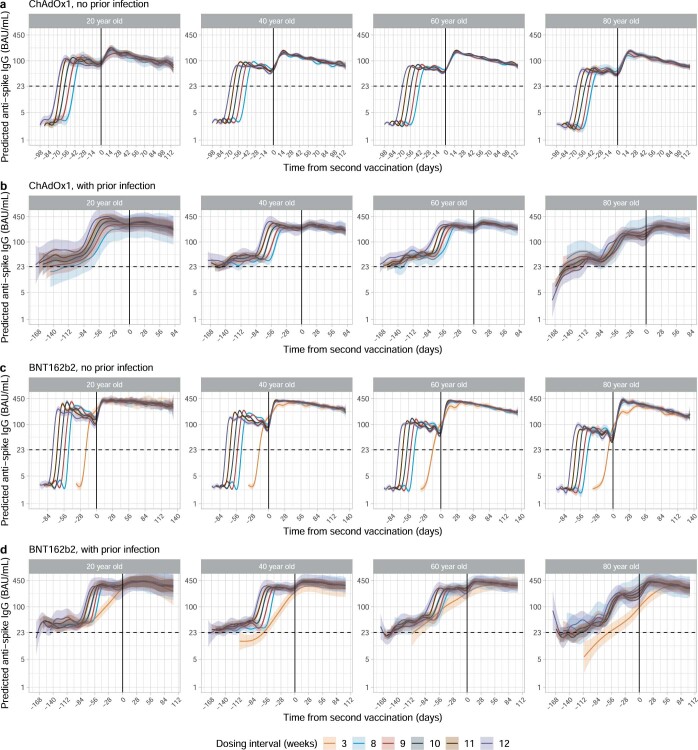
Mean anti-spike IgG levels by time from second vaccination in 20-, 40-, 60- and 80-year-olds according to dosing interval using generalised additive models adjusted for age and dosing interval. **a**, Participants who received two doses of ChAdOx1 without prior infection. **b**, Participants who received two doses of ChAdOx1 with prior infection. **c**, Participants who received two doses of BNT162b2 without prior infection. **d**, Participants who received two doses of BNT162b2 with prior infection. Different *x* axis scales reflect different durations of follow-up post-vaccination in the different cohorts. Predicted levels are plotted on a log scale. Black dotted line indicates the threshold of IgG positivity (23 BAU/mL). Black solid line indicates the second vaccination date. Line colour indicates response predicted for 3 weeks, 8–12 weeks dosing interval. The 95% CIs are calculated by prediction ± 1.96 × standard error of the prediction. Source data

**Extended Data Fig. 3:**
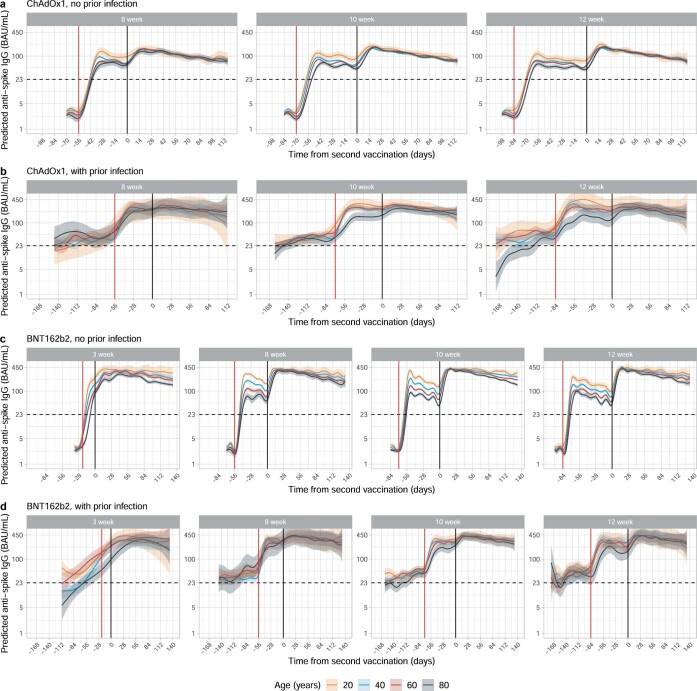
Mean anti-spike IgG levels by time from second vaccination in 3-, 8-, 10-, and 12-week dosing interval groups according to age using generalised additive models adjusted for age and dosing interval. **a**, Participants who received two doses of ChAdOx1 without prior infection. **b**, Participants who received two doses of ChAdOx1 with prior infection. **c**, Participants who received two doses of BNT162b2 without prior infection. **d**, Participants who received two doses of BNT162b2 with prior infection. Different *x* axis scales reflect different durations of follow-up post-vaccination in the different cohorts. Predicted levels are plotted on a log scale. Black dotted line indicates the threshold of IgG positivity (23 BAU/mL). Red solid line indicates the first vaccination and black solid line indicates the second vaccination. Line colour indicates response predicted for age 20, 40, 60, and 80 years. The 95% CIs are calculated by prediction ± 1.96 × standard error of the prediction. Source data

In participants receiving two vaccinations without prior infection, generalized additive models (GAMs) adjusting only for age and dosing interval showed generally similar antibody trajectories for both vaccines but with higher antibody levels achieved with BNT162b2 versus ChAdOx1 (Extended Data Fig. 2a,c and Supplementary Table 2a; observed data shown in Supplementary Fig. 1). Anti-spike IgG levels increased after the first dose, peaked ~21 days later and then gradually declined until the second dose, after which they reached even higher peak levels ~21 days later and then gradually declined again. After the first dose, peak levels were lower in older participants, but age differences were attenuated after the second dose (Extended Data Fig. 3a,c and Supplementary Table 2b). In these minimally adjusted analyses (adjusted for age and dosing interval), there was no evidence of differences in antibody levels and declines after the second dose across 8–12-week dosing intervals for both ChAdOx1 and BNT162b2 vaccines. However, the antibody trajectories were different for the 3-week BNT162b2 dosing interval, where antibody levels gradually increased from the start of the first dose until around 42 days after the second dose, after which antibody levels were generally similar to those with 8–12-week dosing intervals, although slightly lower in 80-year-old participants (Extended Data Fig. 2c).

**Fig. 1:**
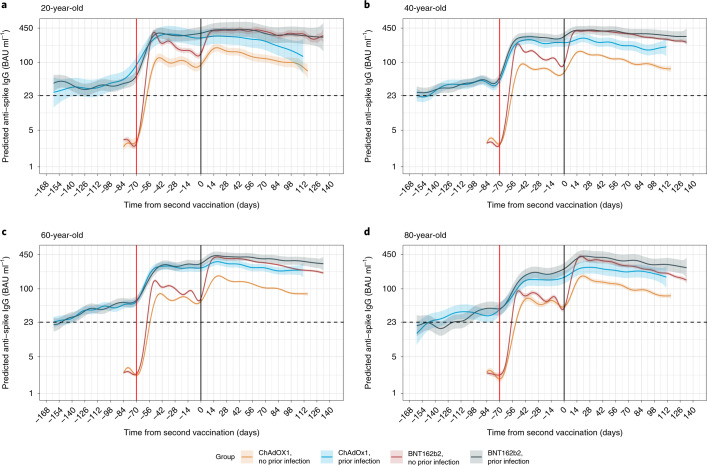
Predicted mean anti-spike IgG levels in participants with 10-week dosing interval by time from second vaccination according to vaccine type, age and prior infection status using GAMs adjusted for age and dosing interval. **a**, 20-year-old. **b**, 40-year-old. **c**, 60-year-old. **d**, 80-year-old. Predicted levels are plotted on a log scale. Black dotted line indicates the threshold of IgG positivity (23 BAU ml^−1^); red solid line indicates the first vaccination; black solid line indicates the second vaccination. Line color indicates response predicted for ChAdOx1 and BNT162b2, with or without prior infection. See Extended Data Fig. 3 for a re-plotted version of the same estimates to allow comparison by age for each vaccine type and prior infection status. See Extended Data Fig. 4 for 8-week and 12-week dosing intervals. The 95% CIs are calculated by prediction ± 1.96 × standard error of the prediction. Source data

**Extended Data Fig. 4:**
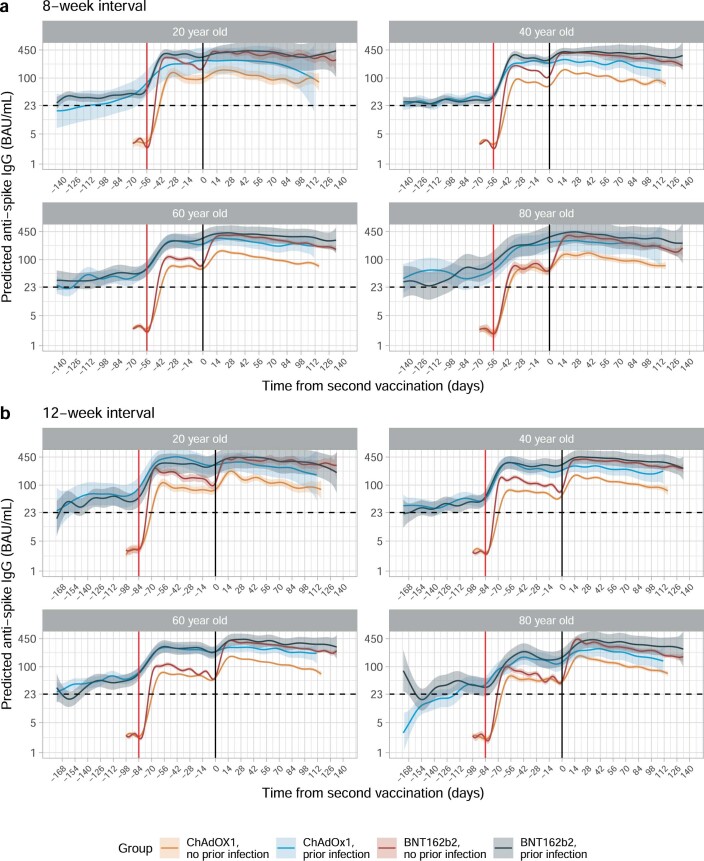
Mean anti-spike IgG levels by time from second vaccination according to vaccine type, prior infection status, and age using generalised additive models adjusted for age and dosing interval. **a**, Participants with a 8-week dosing interval. **b**, Participants with a 12-week dosing interval. Predicted levels are plotted on a log scale. Black dotted line indicates the threshold of IgG positivity (23 BAU/mL). Red solid line indicates the first vaccination and black solid line indicates the second vaccination. Line colour indicates response predicted for ChAdOx1 and BNT162b2, with or without prior infection. The 95% CIs are calculated by prediction ± 1.96 × standard error of the prediction. Source data

In participants with evidence of prior infection, antibody levels started from levels above 23 binding antibody units (BAU) per milliliter (the positivity threshold; Methods) and gradually increased for both vaccines. There was no evidence of differences in antibody levels and declines after the second dose for dosing intervals from 8 to 12 weeks (Extended Data Fig. 2b,d; observed data shown in Supplementary Fig. 2). After prior infection, antibody levels rose to lower levels in older versus younger participants after the first dose, but the difference was attenuated after the second dose (Extended Data Fig. 3b,d). In those with prior infection, there was less boosting seen from the second dose given higher levels after one dose compared to those not previously infected, but there was a second dose boosting effect for participants who were 80 years old (Fig. 1). For ChAdOx1, participants without prior infection had lower antibody levels after the second dose than those with prior infection after the first dose; but, for BNT162b2, two vaccinations without prior infection led to higher antibody levels than previously infected participants having only one dose, especially for 80-year-old participants (Fig. 1; trajectories for other dosing intervals in Extended Data Fig. 4).

### Determinants of anti-spike IgG peak levels and half-life after the second dose

#### ChAdOx1

**Fig. 2:**
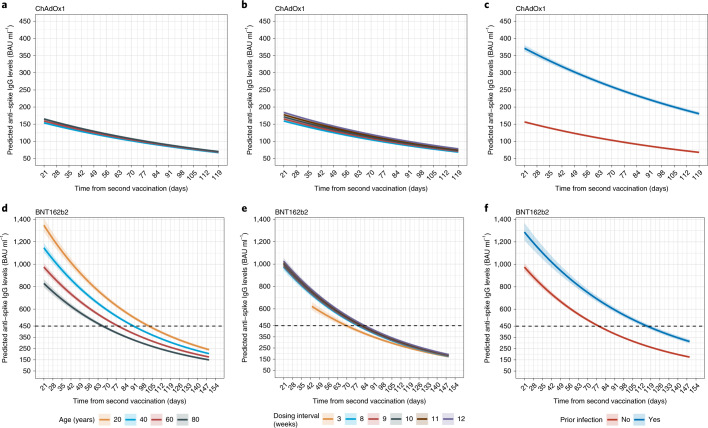
Posterior predicted mean trajectories (95% CrI) of anti-spike IgG levels from 21 days after the second dose using Bayesian linear mixed interval censored models. Models are adjusted for age, sex, ethnicity, long-term health conditions, healthcare role, deprivation, dosing interval and prior infection status. **a**,**d**, Trajectories by age. **b**,**e**, Trajectories by dosing interval. **c**,**f**, Trajectories by prior infection status. **a**–**c**, Participants who received two doses of ChAdOx1. **d**–**f**, Participants who received two doses of BNT162b2. Black dotted line shows the upper quantification limit of 450 BAU ml^−1^. For BNT162b2, as 52% of measurements were above the upper limit, the estimated peak levels were higher than 450 BAU ml^−1^ (interval censoring accounted for in analysis). Plotted at reference categories: 60 years, female, white ethnicity, not reporting a long-term health condition, not a healthcare worker, deprivation percentile of 60, 8-week dosing interval and no prior infection. In **a**, 20-year-old group is not plotted because the vast majority of those receiving ChAdOx1 were 40 years of age or older. These model estimates more completely adjust for confounders than the GAMs plotted in Extended Data Fig. 2 but estimate trends only after second vaccination. Source data

**Extended Data Fig. 5:**
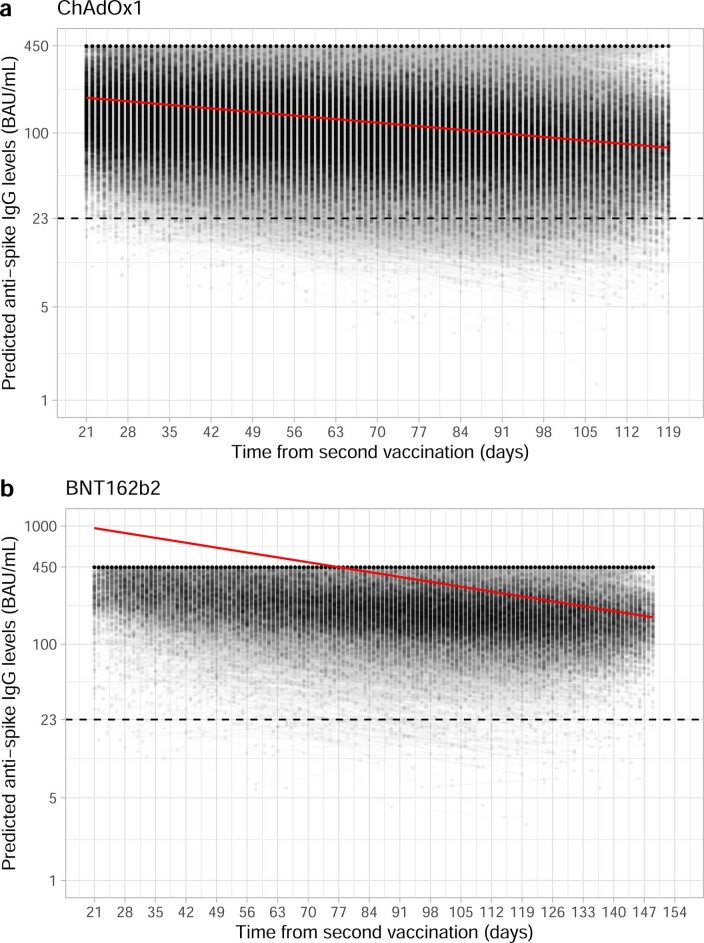
Estimated unadjusted mean trajectory of anti-spike IgG antibody levels and individual trajectories from 21 days after the second vaccination. **a**, In participants who received two doses of ChAdOx1. **b**, In participants who received two doses of BNT162b2. Black dashed line indicates the assay threshold for IgG positivity (23 BAU/mL). For BNT162b2, interval censored regression takes account of the fact that 52% measurements were above the upper quantification limit of 450 BAU/mL, and the timing of these measurements, so the estimated peak levels were higher than 450 BAU/mL. Source data

Of the 133,388 participants (with or without prior infection) who received two ChAdOx1 doses, 100,639 participants contributed 191,137 antibody measurements at least 21 days after the second dose, median (IQR) [range] 2 (1–2) [1–5] measurements per participant (Supplementary Table 3). Antibody measurements were taken a median (IQR) [range] 61 (41–83) [21–119] days after second vaccination (Supplementary Table 3 and Supplementary Fig. 3). Assuming that antibody levels declined exponentially, using Bayesian linear mixed models we estimated a mean peak anti-spike IgG level of 184 BAU ml^−1^ (95% credible interval (Crl), 183–185) and a mean half-life of 79 days (78–80) (Extended Data Fig. 5) versus 113 BAU ml^−1^ (106–117) and 184 days (163–210), respectively, after natural infection before vaccination^16^. There was no evidence that rates of antibody decline flattened over time (up to 119 days after second vaccination; Methods). In a multivariable model, all factors considered (age, sex, ethnicity, reporting a long-term health condition, healthcare work, deprivation, dosing interval and prior infection status) were independently associated with anti-spike IgG peak levels 21 days after the second dose, but most effects were small (Fig. 2 and Supplementary Table 4). The largest effects were associated with prior infection, peak 219 BAU ml^−1^ higher (210–227), and ethnicity, peak 41 BAU ml^−1^ (36–47) higher in those reporting non-white ethnicity. Peak levels were slightly lower in males, in those reporting a long-term health condition, in those not working in healthcare and in those with shorter dosing intervals, younger age and less deprivation (higher deprivation percentile). Prior infection extended the half-life by 13 days (95% Crl, 9–17). There were very small reductions in half-life at older ages, in non-white ethnicity and in having a long-term health condition. There was no evidence of associations between half-life and sex, being a healthcare worker and dosing interval in participants who received ChAdOx1.

#### BNT162b2

In 89,105 participants (with or without prior infection) who received two BNT162b2 doses, 55,053 participants contributed 120,728 antibody measurements at least 21 days after the second dose (≥42 days for those with 3-week dosing interval; Methods and Extended Data Fig. 2), median (IQR) [range] 2 (1–3) [1–6] per participant (Supplementary Table 3). Antibody measurements were taken a median (IQR) [range] 79 (51–106) [21–149] days after the second vaccination (Supplementary Table 3 and Supplementary Fig. 3). The estimated mean peak level was 959 BAU ml^−1^ (95% Crl, 944–974), and the mean half-life was 51 days (50–52) (Extended Data Fig. 5). There was, again, no evidence of antibody decline flattening on the log-scale (up to 149 days after the second vaccination; Methods). Factors had greater effects on peak levels for BNT162b2 than ChAdOx1 (Fig. 2 and Supplementary Table 4). Peak levels were lower at older ages (76 BAU ml^−1^ lower per 10-years older, 95% Crl, 68–84), in males (140 BAU ml^−1^ lower, 117–164) and in those reporting long-term health conditions (79 BAU ml^−1^ lower, 55–104) and were higher in those reporting non-white ethnicity (141 BAU ml^−1^ higher, 78–208), in those working in healthcare (287 BAU ml^−1^ higher, 221–358) and in those who were less deprived (5 BAU ml^−1^ higher per ten percentiles higher, 1–10). However, these factors had little or no effect on half-life. Within dosing intervals between 8 and 12 weeks, longer dosing intervals were associated with higher peak levels (12 BAU ml^−1^ higher per week longer, 1–23) but had no effect on half-life. Compared to an 8-week extended schedule, a 3-week dosing interval was associated with a lower peak level 42 days after the second dose but a slightly longer half-life (6 days longer, 2–10), leading to similar antibody levels at 149 days across different dosing groups. Similarly to ChAdOX1, prior infection was associated with a much higher peak level (312 BAU ml^−1^ higher, 248–381) and a longer half-life (11 days longer, 8–15).

**Fig. 3:**
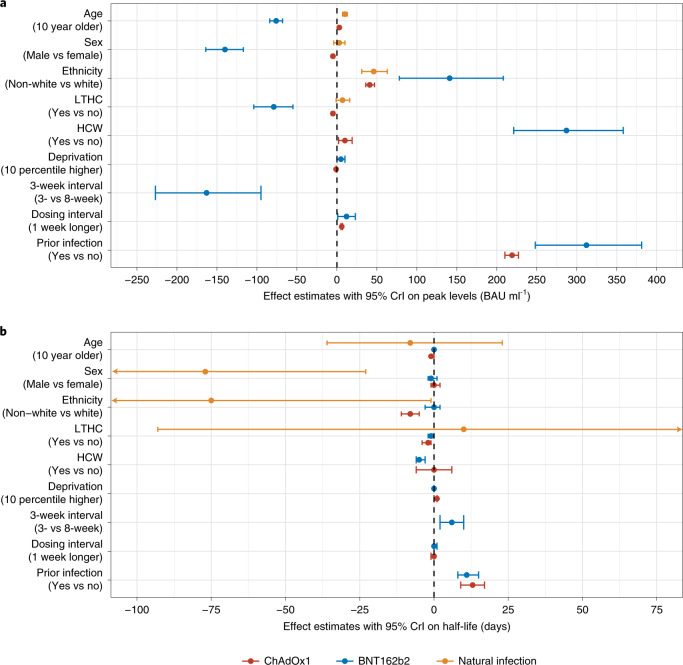
Comparison of effects of factors in participants who received two doses of ChAdOx1 or BNT162b2 or had natural SARS-CoV-2 infection. **a**, Effects on anti-spike IgG peak levels. **b**, Effects on anti-spike IgG half-lives. In total, 100,639 participants received two doses of ChAdOx1; 55,053 participants received two doses of BNT162b2; and 3,271 had natural infection. Mean estimates with 95% CrIs are presented. In **b**, 95% CrIs are truncated at −100 and 75 days for visualization. Source data

Comparing the effects of factors between the two vaccines, and with our previous findings on natural infection^16^ (Fig. 3), effects of some factors were relatively consistent among ChAdOx1, BNT162b2 and/or natural infection, albeit with differing effect sizes (for example, sex, ethnicity, long-term health condition, working in healthcare, dosing interval on peak and prior infection on peak and half-life), whereas, for others, effects were in opposite directions (for example, age and deprivation on peak) or associated with only one vaccine (for example, ethnicity and working in healthcare on half-life). In general, effects on peak levels were greater for BNT162b2 than for ChAdOx1; and, other than prior infection, effects on half-life were limited for both vaccines.

### Correlates of protection

To interpret anti-spike IgG levels over time, we investigated whether recent antibody levels are correlates of protection from infection after prior infection or vaccination in the general population. We used data from 17 May 2021 to 4 October 2021—that is, while the Delta variant accounted for nearly all cases^17^—and fitted logistic GAMs for detected new infections, investigating the effect of the most recent antibody measurement obtained 21–59 days earlier on polymerase chain reaction (PCR) test results at each study visit (distribution of visits relative to first vaccination is shown in Supplementary Fig. 4 and Supplementary Table 5). Three groups were investigated: (1) unvaccinated participants with or without evidence of prior infection (6,833 participants, 12,560 visits); (2) participants vaccinated with ChAdOx1 at least 21 days previously (83,924 participants, 221,380 visits, 36–273 days after the first vaccination); and (3) participants vaccinated with BNT162b2 at least 21 days previously (49,820 participants, 124,822 visits, 35–298 days after the first vaccination). Participants tested PCR-positive at 202 (1.6%), 1,327 (0.6%) and 591 (0.5%) visits, respectively. Vaccinated participants with evidence of prior infection were excluded, as there were insufficient data to model these groups separately, and associations might differ. Adjustment was made for confounders, including age, geography and calendar time (Methods).

**Fig. 4:**
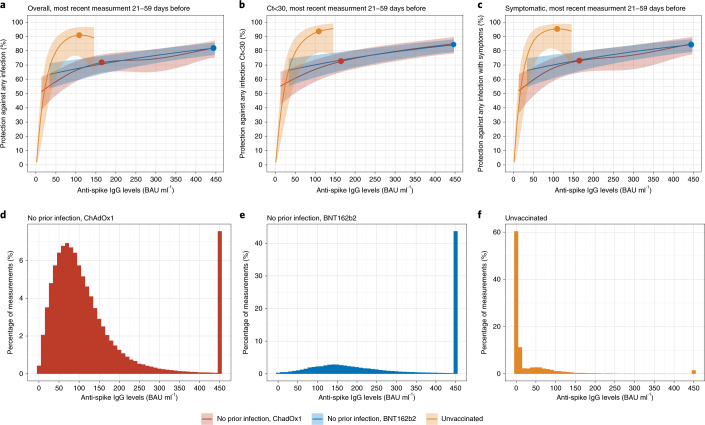
Association between anti-spike IgG levels and protection from SARS-CoV-2 infection using the most recent antibody measurement obtained 21–59 days before the current visit. **a**, Protection against any infection. **b**, Protection against infection with a moderate to high viral load (Ct value < 30). **c**, Protection against infection with self-reported symptoms. The 95% CIs are calculated by prediction ± 1.96 × standard error of the prediction. Three groups are investigated: unvaccinated participants with or without evidence of prior infection, participants vaccinated with ChAdOx1 without evidence of prior infection and participants vaccinated with BNT162b2 without evidence of prior infection. Dots represent the median predicted individual peak levels from the Bayesian linear mixed models: 1,026 BAU ml^−1^ for BNT162b2 (plotted at the upper quantification limit of 450 BAU ml^−1^); 167 BAU ml^−1^ for ChAdOx1; and 111 BAU ml^−1^ for unvaccinated participants^16^. Distribution of the most recent anti-spike IgG measurements for the three population groups are shown in **d**–**f**. See Supplementary Fig. 4 and Supplementary Table 5 for timing of visits relative to first vaccination. Source data

**Extended Data Fig. 6:**
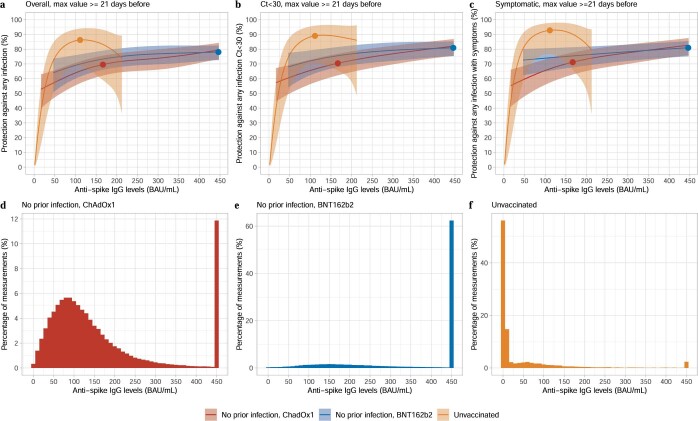
Association between anti-spike IgG levels and protection from SARS-CoV-2 infection using the maximum antibody measurement obtained ≥21 days prior to the visit. **a**, protection against any infection; **b**, protection against infection with a moderate to high viral load (Ct value <30); **c**, protection against infection with self-reported symptoms. The 95% CIs are calculated by prediction ± 1.96 × standard error of the prediction. Three groups are investigated, unvaccinated participants with or without evidence of prior infection, participants vaccinated with ChAdOx1 without evidence of prior infection, and participants vaccinated with BNT162b2 without evidence of prior infection. Dots represent the median predicted individual peak levels from the Bayesian linear mixed models: 1026 BAU/mL for BNT162b2 (plotted at the upper quantification limit of 450 BAU/mL), 167 BAU/mL for ChAdOx1, and 111 BAU/mL for unvaccinated participants^1^. Distribution of the maximum anti-spike IgG measurements for the three population groups are shown in panel **d, e, f**. See Supplementary Fig. 4 and Supplementary Table 5 for timing of visits relative to second vaccination. Source data

Compared to unvaccinated individuals with a most recent anti-spike IgG measurement of 1 BAU ml^−1^, protection against infection increased steeply as antibody levels rose in unvaccinated participants, consistent with protection from prior infection, with 50% protection at 20 BAU ml^−1^ (below the 23 BAU ml^−1^ positivity threshold) and 67% protection at 33 BAU ml^−1^. Higher antibody levels were needed to achieve the same level of protection after vaccination, with no evidence of differences in protection between ChAdOx1 and BNT162b2 at any given antibody level (Fig. 4a). For example, considering antibody levels where two-thirds (67%) of individuals were protected against infection, participants vaccinated with ChAdOx1 or BNT162b2 required estimated levels of 107 BAU ml^−1^ and 94 BAU ml^−1^ (100 BAU ml^−1^ in models pooling both vaccines), respectively. However, as described above, antibody levels rose to higher levels after BNT162b2 than after ChAdOx1, explaining higher vaccine effectiveness after BNT162b2 versus after ChAdOx1 (ref. ^18^), with natural infection resulting in the lowest antibody levels but the greatest protection at a given antibody level (Fig. 4d–f). Protection against infection with moderate to high viral loads (cycle threshold (Ct) values < 30) (Fig. 4b) and symptomatic infection (Fig. 4c) was similar. Findings were also similar when considering the maximum prior antibody measurement (which had a worse model fit), in part because of the limited time for antibody waning to occur between maximum and most recent antibody measurements (Extended Data Fig. 6).

### Duration of antibody response and association with protection

**Fig. 5:**
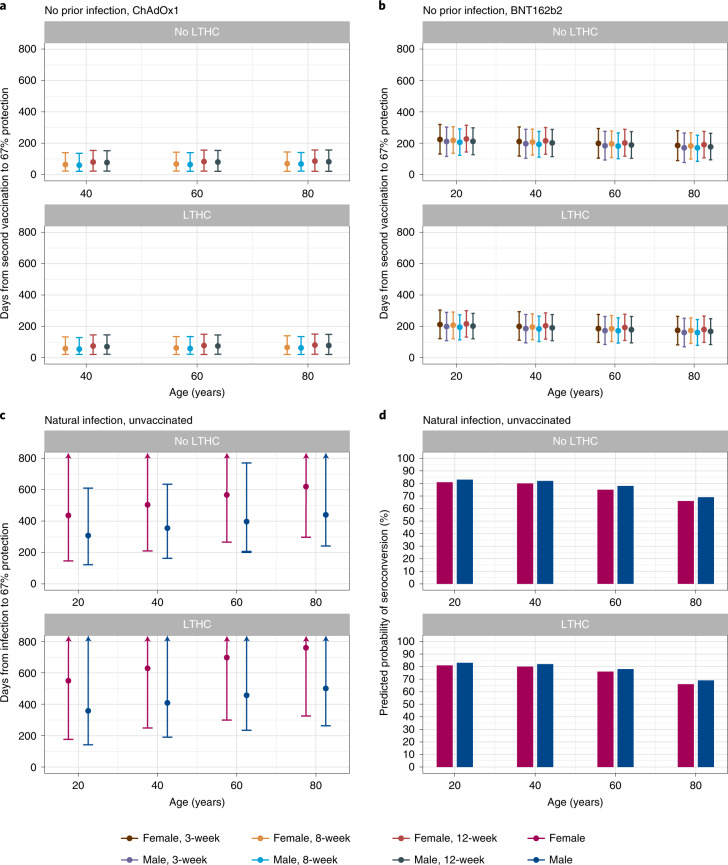
Posterior predicted mean days (95% CrI) from the second vaccination/infection to the threshold level associated with 67% protection. The threshold level is 107 BAU ml^−1^ for ChAdOx1, 94 BAU ml^−1^ for BNT162b2 and 33 BAU ml^−1^ for unvaccinated. **a**, In participants without prior infection and vaccinated with ChAdOX1 (*n* = 92,584). **b**, In participants without prior infection and vaccinated with BNT162b2 (*n* = 51,034). **c**, In unvaccinated participants who had natural infection (*n* = 3,271). **d**, Predicted probability of seroconverting in unvaccinated participants who had natural infection, based on a previous model on seroconversion^16^. Estimates were separated by age, sex, dosing interval, long-term health condition (LTHC) and vaccine type for vaccinated people and by age, sex and LTHC for unvaccinated people. *y* axis is truncated at 800 days for visualization. For ChAdOx1, 20-year-old group is not plotted because the vast majority of those receiving ChAdOx1 were 40 years of age or older. **a**–**c** are conditional on participants having antibody response/seroconverting (see the ‘Vaccine non-responders’ section for ChAdOx1 and BNT162b2 and discussion in a previous publication^16^ for unvaccinated individuals). Source data

**Extended Data Fig. 7:**
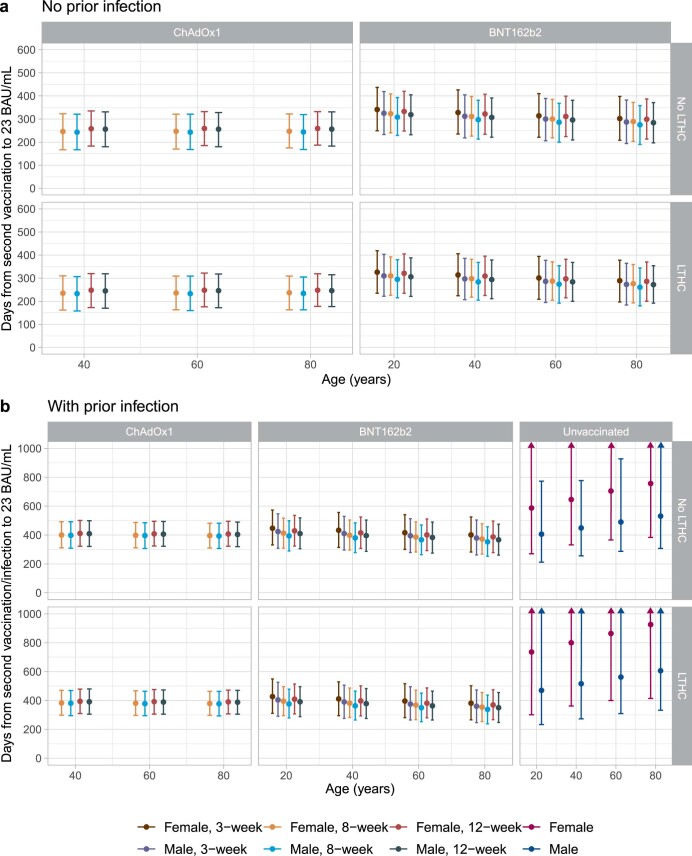
Posterior predicted mean days (95% credible interval) from the second vaccination/infection to the positivity threshold of 23 BAU/mL. **a**, In those without evidence of prior infection, 92,584 participants vaccinated with ChAdOx1, 51,034 participants vaccinated with BNT162b2. **b**, In those with evidence of prior infection, 8,055 vaccinated with ChAdOx1, 4,019 vaccinated with BNT162b2, 3,271 had natural infection and unvaccinated. Estimates were separated by age, sex, dosing interval, long-term health condition (LTHC), and vaccine type for vaccinated people, and by age, sex, and LTHC for unvaccinated people. y-axis is truncated at 1000 days (panel b) for visualisation. For ChAdOx1, 20-year-old group is not plotted because the vast majority of those receiving ChAdOx1 were ≥40 years. Estimates for the unvaccinated group in panel b are based on a previously reported biphasic model assuming the rate of antibody decline slowing over time, which may explain the longer point estimates vs ChAdOx1 or BNT162b2, however credible intervals also overlap. Source data

Models of correlates of protection allowed post-vaccination antibody levels corresponding to 67% protection from Delta variant infection to be estimated (data were insufficient to estimate lower protection percentages for BNT162b2). The estimated mean time from second vaccination to antibody measurements reaching levels associated with 67% protection was 55–86 days for ChAdOx1-vaccinated participants without prior infection, with limited variation across age, sex, dosing interval or long-term health conditions (Fig. 5a). For BNT162b2, the estimated mean durations were 161–227 days and were longer at younger ages, in females and in those without long-term health conditions but were similar across different dosing intervals (Fig. 5b). For unvaccinated participants infected previously, using a model of antibody declines after natural infection^16^, the duration from diagnosis to the level associated with 67% protection was estimated to be 1–2 years (Fig. 5c). Older people had a longer duration of protection after natural infection, but these results were conditional on seroconverting, and older individuals had lower seroconversion rates (Fig. 5d). Although data were insufficient to estimate antibody levels correlated with protection for vaccinated participants with prior infection, conservatively assuming that the threshold levels were similar to those vaccinated without prior infection, and given that their half-lives were longer, the duration of protection could last for more than 1 year. Times for antibody levels to fall to the threshold for positivity—that is, 23 BAU ml^−1^—were longer but followed the same patterns (Extended Data Fig. 7).

**Fig. 6:**
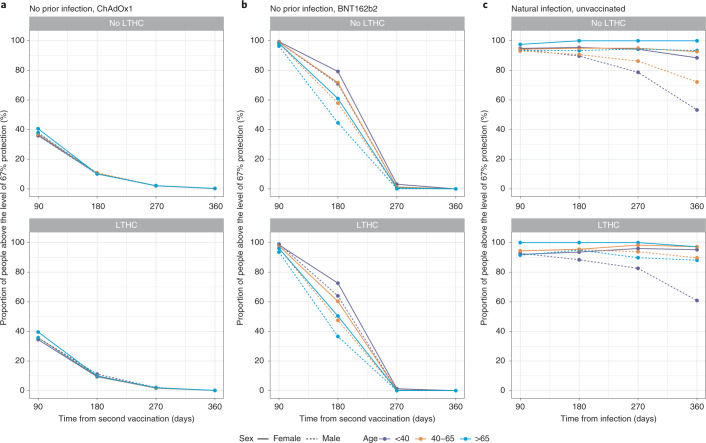
Proportion of participants above the threshold level associated with 67% protection by time from second vaccination/infection. The threshold level is 107 BAU ml^−1^ for ChAdOx1, 94 BAU ml^−1^ for BNT162b2 and 33 BAU ml^−1^ for unvaccinated. **a**, In participants without prior infection and vaccinated with ChAdOX1. **b**, In participants without prior infection and vaccinated with BNT162b2. **c**, In participants who had natural infection and unvaccinated. Estimates were separated by age, sex and long-term health condition (LTHC). Numbers of participants in each panel are in the order of no-LTHC and LTHC; numbers in brackets represent <40, 40–65 and >65: **a**, *n* = 72,121 [4,973, 46,160, 20,988] and 28,518 [2,166, 14,315, 12,037]; **b**, *n* = 36,662 [6,650, 14,108, 15,904] and 18,391 [1,800, 6,696, 9,895]; **c**, *n* = 2,625 [1,207, 1,243, 175] and 646 [194, 322, 130]. All panels are conditional on participants having antibody response/seroconverting (see the ‘Vaccine non-responders’ section for ChAdOx1 and BNT162b2 and discussion in a previous publication^16^ for unvaccinated individuals). Source data

We estimated the proportion of participants with 67% protection at 90, 180, 270 and 360 days from the second dose or natural infection based on individual-level predictions, conditional on seroconverting. At 180 days, 10% of ChAdOx1 participants without prior infection remained above the level associated with 67% protection, with little variation across different factors. For BNT162b2, the proportion varied between 40% and 80% for those without prior infection and was higher in younger ages, in females and in those without long-term health conditions. Over 90% of unvaccinated participants with natural infection were above the level required for 67% protection 180 days after diagnosis. At 270 days, almost everyone receiving ChAdOx1 or BNT162b2 fell below the level of 67% protection, whereas over 80% of unvaccinated participants were still above this threshold level (Fig. 6).

**Extended Data Fig. 8:**
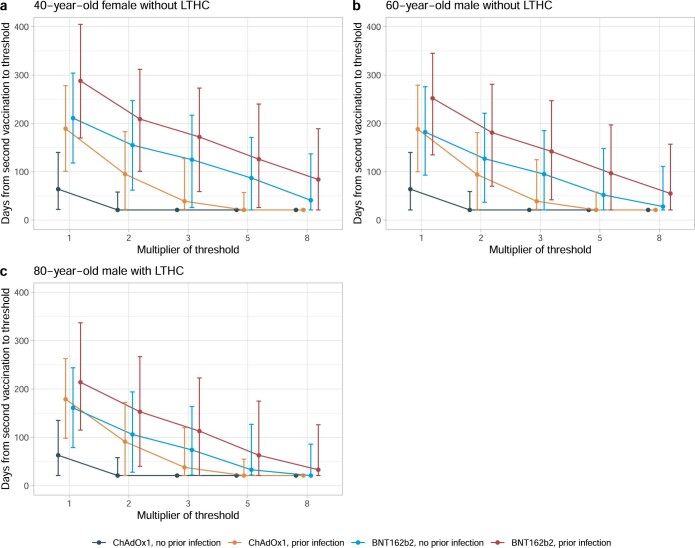
Posterior predicted days (95% credible interval) from the second vaccination to the threshold level associated with 67% protection multiplied by 2, 3, 5, 8, according to prior infection status and vaccine type. The threshold level is 107 BAU/mL for ChAdOx1 and 94 BAU/mL for BNT162b2. **a**, in a 40-year-old female without long-term health conditions. **b**, in a 60-year-old male without long-term health conditions. **c**, in an 80-year-old male with long-term health conditions. Estimates are based on 100,639 participants vaccinated with ChAdOx1 and 55,053 participants vaccinated with BNT162b2. All three panels were plotted at an 8-week dosing interval. LTHC: long-term health condition. Multipliers reflect the fact that higher antibody level may be required for protection against variants of concern. Source data

Emerging viral variants might need higher antibody levels for the same level of neutralizing activity^19^. In a sensitivity analysis assuming two-fold to eight-fold increases above the threshold associated with 67% protection against the Delta variant, protection from two vaccine doses was short-lived. For example, in a 40-year-old female without any long-term health conditions and without prior infection, if three-fold higher antibody levels were required, BNT162b2 would provide around 120 days of protection, whereas ChAdOx1 did not reach the required antibody level; furthermore, if eight-fold higher antibody levels were required, the duration of protection from BNT162b2 would be reduced to only 40 days (Extended Data Fig. 8).

### Vaccine non-responders

We previously used latent class mixed models to identify 5.8% and 5.1% of a smaller population of participants receiving one ChAdOx1 or BNT162b2 dose, respectively, as non-responders^20^. Because latent class models would not fit with larger numbers, we used a heuristic rule based on these previous observations to define non-response as all antibody measurements less than 16 BAU ml^−1^ (similar levels to the previous non-response class) and having at least one antibody measurement 21 days after the first or second dose. To examine robustness, we also restricted to those having at least two antibody measurements and after both doses (rather than each separately). Across different assumptions (Supplementary Table 6), we found that 5.8–7.7% and 3.5–6.0% of participants were classed as non-responders to the first ChAdOx1 or BNT162b2 dose, respectively, similar to previously. Non-responders were older, had a higher percentage of males and white ethnicity, were more deprived, were less likely to be working in healthcare and were more likely to have long-term health conditions (Supplementary Table 7). However, only 0.4–1.0% and 0.1–0.5% of participants were non-responders to the second ChAdOx1 or BNT162b2 dose, respectively, and 0.3–0.5% and 0.1–0.2% were non-responders to both the first and second doses of ChAdOx1 or BNT162b2, respectively.

## Discussion

Based on a large random sample of the UK population, we found significant boosting of anti-spike IgG after the second doses of both ChAdOx1 and BNT162b2 vaccines in all age groups and using different dosing intervals, including the 3-week dosing interval for BNT162b2. Consistent with our previous findings^20^, those receiving BNT162b2 had significantly higher peak anti-spike IgG responses than those receiving ChAdOx1; however, their antibody levels fell faster than after ChAdOx1. After the second vaccination, older age, male sex and long-term health conditions were all associated with substantially lower peak levels in participants who received BNT162b2 but had smaller effects on peak levels with ChAdOx1. Participants with prior infection had significantly higher peak levels for both vaccines, and their antibody levels fell more slowly. Based on our estimates of anti-spike antibody levels as correlates of protection, antibody levels associated with 67% protection against infection with Delta last for 2–3 months after a second ChAdOx1 dose and for 5–8 months after BNT162b2 in those without prior infection and could last for 1–2 years in those unvaccinated but seroconverting after natural infection. Similar to our previous findings^20^, around 6–8% and 4–6% of participants were non/low-responders who did not substantially increase their antibody levels after the first ChAdOx1 or BNT162b2 dose. However, non-response to a second ChAdOx1 or BNT162b2 dose was much smaller, less than 1%, suggesting that second doses can significantly boost an initial suboptimal response in most individuals.

Antibody trajectories after vaccination differed substantially by prior infection status. Prior infection was associated with a significantly higher peak level and a longer half-life for both ChAdOX1 and BNT162b2. This is consistent with studies in healthcare workers showing that prior SARS-CoV-2 infection leads to higher antibody levels and slower waning after the second BNT162b2 vaccination^21–24^. Previous studies also reported that a single dose of BNT162b2 or Sputnik V, an adenovirus-based vaccine, elicited post-vaccination antibody levels that were similar to, or higher than, those without prior infection who received two doses^25–28^. From the GAMs, we found slightly lower IgG levels after a single BNT162b2 dose in previously infected participants versus those with two BNT162b2 doses without prior infection, particularly at older ages. This suggests that a second BNT162b2 dose might still be helpful for previously infected individuals where supplies are sufficient, especially for older age groups. However, for ChAdOx1, the post-second-dose IgG levels in those without prior infection were lower than in those previously infected and vaccinated with one ChAdOx1 dose.

In an adjusted Bayesian linear mixed model, longer dosing intervals between 8 and 12 weeks resulted in higher peak antibody levels for ChAdOx1 (6 BAU ml^−1^ per week) and BNT162b2 (12 BAU ml^−1^ per week) but had no effect on the half-life. Consistent with this, the 3-week dosing interval for BNT162b2 resulted in a lower peak level compared to an 8-week interval. Other studies also reported lower antibody levels at 2–3 weeks^7^, 4 weeks^8^ and 14–34 days^9^ after the second BNT162b2 dose with 3-week versus extended dosing intervals. However, these studies measured antibody levels only at specific time points after second vaccination, which might not be optimal for comparison given that we found that antibody levels were still increasing from 3–6 weeks after the second dose with the 3-week interval (Extended Data Fig. 2). We found a slightly longer half-life with 3-week versus extended dosing intervals, which led to similar duration of protection against new infections despite the lower peak levels.

Older individuals had lower post-second-dose IgG peak levels after BNT162b2, but age-related differences were smaller for ChAdOx1. Females had a higher peak IgG level for both vaccines, consistent with widely reported enhanced immune responses in females^29–33^. Healthcare workers had higher IgG peak levels for both vaccines, potentially reflecting a ‘healthy worker’ effect^34^, ongoing occupational exposure or undetected prior infection. We also found that those reporting non-white ethnicity had higher IgG peak levels. Non-white ethnicity has also been previously associated with higher antibody levels after natural infection^16,35,36^; these findings could be due to genetic or societal differences or differential rates of undetected prior infection. Long-term health conditions were associated with lower peak levels and shorter half-lives for both vaccines. Our results on BNT162b2 were consistent with a recent large-scale study from Israel, where older individuals, males and those reporting health conditions had significantly lower IgG levels^37^.

Differences were also seen between the two vaccines. For example, ChAdOx1 had a lower peak level, but BNT162b2 had a shorter half-life, and factors generally had larger effects on BNT162b2 than on ChAdOx1. Differences in vaccine response are expected given the differing design and mechanism of action of BNT162b2, an mRNA vaccine, and ChAdOx1, an adenovirus vector-based vaccine^38,39^. Alternative booster vaccines might reduce vaccine-specific differences^40^.

We estimated anti-spike IgG mean half-lives after second ChAdOx1 or BNT162b2 doses of 81 days and 52 days, respectively, in those without prior infection and 94 days and 63 days in those vaccinated and previously infected. The half-life of BNT162b2 was consistent with estimates from previous studies at around 50 days^41–43^. Data from ChAdOx1 and mRNA-1273 vaccine trials showed higher levels of binding, and neutralizing antibodies at a limited number of time points were associated with a lower risk of infection and a higher vaccine efficacy^14,15^. However, correlating time-updated antibody measurements to protection from infection is important to inform the timing of boosters and other control measures, but existing studies combining correlates of protection and longitudinal data from the same assay are limited. We found that higher anti-spike IgG levels were associated with increased protection from infection but that the level of protection associated with a given antibody level depended on the mechanism generating the antibodies, with natural infection resulting in lower measured antibody levels but greater protection at a given antibody level compared to vaccination. We did not see evidence of vaccine-specific differences, with the greater protection from BNT162b2 versus ChAdOx1 explained by higher antibody levels rather than increased protection at a given antibody level. Using 67% protection against infection as an example threshold, protection was relatively short-lived in those not previously infected and receiving ChAdOx1. Mean levels fell below this threshold at 50–90 days after the second dose, but protection was more prolonged after two doses of BNT162b2, 160–230 days. In those without prior infection, 6 months after the second dose, only 10% of those with ChAdOx1 would maintain 67% protection, whereas 40–80% of those with BNT162b2 would still be above the threshold, indicating that a booster dose may be prioritized to those who had two doses of ChAdOx1 and would need to be individualized to optimize protection after two BNT162b2 doses. However, 9 months after the second dose of either vaccine, almost everyone without prior infection was below the threshold level associated with 67% protection. Our results are consistent with several recent studies reporting waning vaccine effectiveness 3–6 months after second BNT162b2 or ChAdOx1 vaccinations^44–48^. Estimated durations of protection were much longer after natural infection, ~1–2 years for those unvaccinated. The protection in those vaccinated with prior infection could be even longer given that they have a longer half-life; however, we were not able to quantify this owing to limited data. Our estimates of correlates of protection were based on Delta variant infections and so account for the increased antibody levels needed for neutralization of this variant compared to earlier variants^49,50^. However, further increases in antibody levels required for protection with other variants, such as Omicron, would substantially reduce the proportion of the population protected from infection. For example, one study found that neutralization of Omicron was eight-fold lower than Delta after two BNT162b2 vaccinations^51^. In the case where relationships between antibody levels and levels of protection have not changed with Omicron, this would mean that antibody levels after two vaccinations would not provide effective protection against Omicron infection (Extended Data Fig. 8). Nevertheless, protection against severe infection is likely to last considerably longer and be potentially more robust^52^. Further long-term follow-up data are essential for ongoing monitoring, as it is difficult to predict the emergence of and level of immune protection required for new variants.

Limitations of this study include the fact that, to predict vaccine effectiveness from antibody levels beyond the study follow-up, we assumed that associations between protection and antibody levels were constant over time. Even though antibody levels decrease over time, other vaccine-induced immune mechanisms, including memory responses, might theoretically provide greater protection at lower antibody levels. There might also be differences in antibody levels and classes in peripheral blood compared to potential exposure sites such as the respiratory tract. Hence, our estimated duration of protection could potentially be underestimated if other immune responses confer longer protection. However, our observations based on waning antibodies are consistent with waning of vaccine effectiveness in our cohort^12^ and other studies^44–48^.

**Extended Data Fig. 9:**
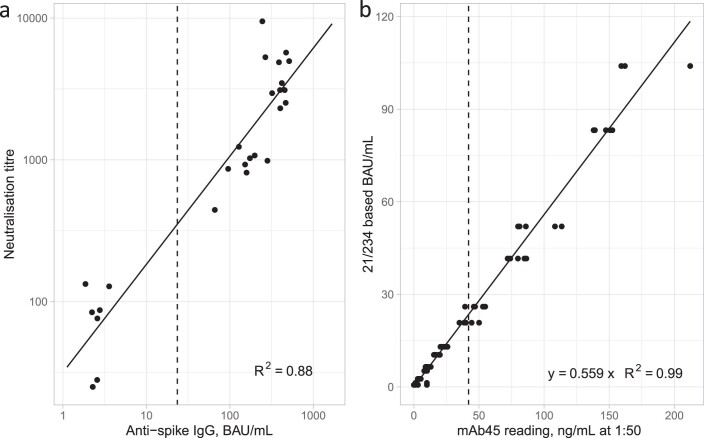
Correlation plots of anti-spike IgG levels. **a**, Correlation between anti-spike IgG levels (BAU/mL) and neutralisation titres in 37 samples. Samples were obtained from the National Institute for Biological Standards and Control (NIBSC, Potters Bar, UK) Anti-SARSCoV-2 Verification Panel for Serology Assays (NIBSC code: 20/B770). **b**, Correlation between anti-spike IgG levels in mAb45 units (ng/ml) and WHO international units (BAU/mL) in 63 dilution replicates. Source data

Given the scale of the study, we did not measure other immune responses, including memory-based responses and T cell or innate immune responses, which are also involved in protection against infection^38^; they might explain the greater protection afforded by natural infection than vaccination at the same antibody level. Other study limitations include insufficient data to model two Moderna mRNA-1273 vaccine doses. We measured anti-spike IgG antibody using a single assay, with the upper limit of quantification reached by 13% and 52% of measurements after the second ChAdOx1 or BNT162b2 vaccination, respectively, potentially leading to underestimating peak levels and overestimating half-lives in those with the highest responses—for example, younger age groups. However, we used interval censored regression models to address this and capture any waning above the threshold, making it possible to estimate a higher peak level than the upper limit and a more accurate half-life. Neutralizing antibodies were not measured, but neutralization titers were strongly correlated with anti-spike IgG titers (Extended Data Fig. 9a). We also calibrated antibody levels to World Health Organization (WHO) BAU ml^−1^ units for comparison with other studies (Extended Data Fig. 9b).

In summary, the second ChAdOx1 or BNT162b2 dose significantly boosts anti-spike IgG levels, and dosing interval has a limited effect on antibody response. Peak levels were higher after BNT162b2 than after ChAdOx1, but older individuals, males and those with long-term health conditions have lower antibody levels with BNT162b2. Protection based on the threshold level associated with 67% protection can last for 2–3 months for ChAdOx1 and for 5–8 months for BNT162b2 in those without prior infection and can be 1–2 years for those unvaccinated who seroconvert after natural infection. Those vaccinated with prior infection could be protected for more than 1 year. These results might inform vaccination strategies. A third boosting dose should be prioritized to ChAdOx1 recipients, to groups with faster antibody declines and to more clinically vulnerable individuals.

## Methods

### Population and setting

The UK’s Office for National Statistics COVID-19 Infection Survey (ISRCTN21086382) randomly selects private households on a continuous basis from address lists and previous surveys to provide a representative sample across its four countries (England, Wales, Northern Ireland and Scotland). After obtaining verbal agreement to participate, a study worker visited each household to take written informed consent from individuals 2 years of age or older. At the first visit, participants were asked for consent for optional follow-up visits every week for the next month and then monthly for 12 months or to April 2022. This consent was obtained from parents/carers for participants 2–15 years of age; those 10–15 years of age also provided written assent. Children younger than 2 years of age were not eligible for the study. For the current analysis, we only included participants aged 16 years or older who were eligible for vaccination for most of the study period.

Individuals were surveyed on their socio-demographic characteristics, behaviors and vaccination status. Combined nose and throat swabs were taken from all consenting household members for SARS-CoV-2 PCR testing. For a random 10–20% of households, individuals 16 years of age or older were invited to provide blood samples monthly for serological testing. Household members of participants who tested positive were also invited to provide blood monthly for follow-up visits. Details on the sampling design are provided elsewhere^53^. From April 2021, additional participants were invited to provide blood samples monthly to assess vaccine responses, based on a combination of random selection and prioritization of those in the study for the longest period (independent of test results). The study protocol is available at https://www.ndm.ox.ac.uk/covid-19/covid-19-infection-survey/protocol-and-information-sheets. The study received ethical approval from the South Central Berkshire B Research Ethics Committee (20/SC/0195).

From 8 December 2020 to 4 October 2021, 402,348 participants 16 years of age or older received two ChAdOx1 or BNT162b2 vaccinations. The median (IQR) age was 56 (41–68) years; 216,301 (53.8%) were females; and 375,880 (93.4%) reported white ethnicity. The median (IQR) deprivation percentile was 62 (38–82), and dosing interval was 74 (63–78) days. In total, 11,565 (2.9%) reported working in patient-facing healthcare, and 112,074 (27.9%) reported having a long-term health condition. Among those 402,348 participants, following the design described above with restricted blood sampling, 222,493 participants gave at least one blood sample for antibody testing from 91 days before the first vaccination onward and were included in our analyses. Participant characteristics were similar between those who did not give blood samples and those who gave blood samples for antibody measurements and were similar across participants with different numbers of antibody measurements (1–2, 3–4 and ≥5) (Supplementary Table 8).

### Vaccination data

Vaccination information was obtained from participants at visits by self-report, including vaccination type, number of doses and vaccination dates. Participants from England were also linked to the National Immunisation Management Service (NIMS), which contains all individuals’ vaccination data in the English National Health Service COVID-19 vaccination program. There was good agreement between self-reported and administrative vaccination data (98% on type and 95% on date^18^). We used vaccination data from NIMS where available for participants from England and otherwise data from the survey.

Participants aged 16 years or older who received two doses of ChAdOx1 or BNT162b2 from 8 December 2020 onward with antibody measurements from 91 days before the first vaccination date up until 4 October 2021 were included in the main analysis. Only 4,219 participants received two doses of mRNA-1273 and, thus, were not included (Extended Data Fig. 1).

### Laboratory testing

SARS-CoV-2 antibody levels were measured on venous or capillary blood samples using an ELISA detecting anti-trimeric spike IgG developed by the University of Oxford^53,54^. Normalized results are reported in ng ml^−1^ of mAb45 monoclonal antibody equivalents. Before 26 February 2021, the assay used fluorescence detection as previously described, with a positivity threshold of 8 million units validated on banks of known SARS-CoV-2-positive and SARS-CoV-2-negative samples^54^. After this, it used a commercialized CE-marked version of the assay, the OmniPATH 384 Combi SARS-CoV-2 IgG ELISA (Thermo Fisher Scientific), with the same antigen and colorimetric detection. mAb45 is the manufacturer-provided monoclonal antibody calibrant for this quantitative assay. To allow conversion of fluorometrically determined values in arbitrary units, we compared 3,840 samples that were run in parallel on both systems. A piece-wise linear regression was used to generate the following conversion formula:

$$log10\left( {mAb45\,units} \right) = 0.221738 + 1.751889e - 07 \ast fluorescence\_units +$$

$$\begin{array}{rcl}5.416675e - 07 \ast \left( {fluorescence\_units > 9190310} \right)\\ \ast \left( {fluorescence\_units - 9190310} \right)\end{array}$$

Each batch of 320 samples (diluted 1:50) was run with a negative control sample (Sigma human serum H6194, diluted 1:50) included in duplicate and a dilution series of three monoclonal antibodies^55–57^ included in duplicate used for assay calibration and quality control (CR3022—four dilutions (1,000, 300, 100 and 30 ng ml^−1^), mAb45—five dilutions (400, 300, 100, 30 and 10 ng ml^−1^) and mAb269—four dilutions (300, 100, 30 and 10 ng ml^−1^)). Values obtained for each control and calibration sample were compared to established historic control values, and plates were subjected to acceptance criteria that required all 28 controls and calibrants to fall within historic limits—namely, no more than five control samples >2 standard deviations different; no more than two samples >3 standard deviations different; and no more than one sample >4 standard deviations different. The first two limits were based on rejecting batches where the probability of the observed variation excluded that expected 99% of the time. The latter rule allowed for one-off robotic error.

We calibrated the results of the OmniPATH assay into WHO international units (BAU ml^−1^) using serial dilutions of National Institute for Biological Standards and Control (NIBSC) Working Standard 21/234. The NIBSC 21/234 Working Standard was previously calibrated against the WHO International Standard for anti-SARS-CoV-2 immunoglobulin (NIBSC code 20/136), with anti-spike IgG potency of 832 BAU ml^−1^ (95% confidence interval (CI), 746–929). We generated two-fold dilutions of 21/234 between 1:400 and 1:8,000 from three separate batches on three separate days. Results from a total of 63 diluted samples were merged, and a linear regression model was fitted constrained to have an intercept of zero to convert mAB45 units in ng ml^−1^ for samples diluted at 1:50 to BAU ml^−1^ (Extended Data Fig. 9b):

$${{{\mathrm{BAU}}}}/{{{\mathrm{mL}}}} = 0.559 \ast \left[ {{{{\mathrm{mAb}}}}45\,{{{\mathrm{concentration}}}}\,{{{\mathrm{in}}}}\,{{{\mathrm{ng}}}}/{{{\mathrm{mL}}}}\,{{{\mathrm{at}}}}\,1:50} \right]$$

We used ≥23 BAU ml^−1^ as the threshold for determining IgG positivity (corresponding to the 8 million units with fluorescence detection). Given the lower and upper limits of the assay, measurements <1 BAU ml^−1^ (2,922 observations, 0.4%) and >450 BAU ml^−1^ (146,337 observations, 19.2%) were truncated at 1 BAU ml^−1^ and 450 BAU ml^−1^, respectively.

Combined nose and throat swabs were tested by PCR assays using the TaqPath SARS-CoV-2 assay (Thermo Fisher Scientific) at high-throughput national ‘Lighthouse’ laboratories in Glasgow and Milton Keynes (up until 8 February 2021). PCR outputs were analyzed using FastFinder 3.300.5 (UgenTec), with an assay-specific algorithm and decision mechanism that allows conversion of amplification assay raw data into test results with minimal manual intervention. Positive samples are defined as having at least a single *N*-gene and/or *ORF1ab* detected (although *S*-gene Ct values are determined, *S*-gene detection alone is not considered sufficient to call a sample positive^53^) and PCR traces exhibiting an appropriate morphology.

### Statistical analysis

Analysis of antibody levels included participants aged 16 years or older who received two doses of ChAdOx1 or BNT162b2 vaccines with or without prior SARS-CoV-2 infection. Age was truncated at 85 years in all analyses to reduce the influence of outliers. Prior infection was defined as having a PCR-positive swab test recorded in the survey or the English national testing program (national testing data were not available for Scotland, Wales and Northern Ireland) or a prior positive anti-spike IgG result (≥23 BAU ml^−1^) any time before the first vaccination. Where participants were known to have become infected after vaccination (based on positive PCR tests from the survey or national linked data), antibody measured after infection was excluded from the analyses. The dosing interval was calculated from the first and second vaccination dates. For the main analysis, we excluded a small number of participants who were considered non-responders after the first or second dose, which was defined as all antibody measurements being <16 BAU ml^−1^ and having at least one antibody measurement 21 days after the first or second dose (*n* = 5,098 excluded for ChAdOx1 and *n* = 1,649 excluded for BNT162b2) (Extended Data Fig. 1). We also excluded participants with recorded dosing interval <49 days or >91 days for ChAdOX1 (*n* = 4,748 excluded) and 29–48 days or >91 days for BNT162b2 (*n* = 6,374 excluded). 17–28 days were classified as a 3-week interval for BNT162b2 and were included.

We used linear GAMs to model anti-spike IgG antibody measurements after the first and second dose to identify the most appropriate time points to model antibody declines after the second vaccination in more detail. Antibody measurements truncated at 450 BAU ml^−1^ were counted as 450 BAU ml^−1^. We built separate models by vaccine type and prior infection status given the hypothesis that antibody response would vary by these two factors. Each model was minimally adjusted for only age and dosing interval using a tensor product of B-splines to allow for non-linearity and interaction among age, dosing interval and time since vaccination, setting the date of the second vaccination as *t* = 0. The smoothing penalty was selected using fast restricted maximum likelihood as implemented in the mcgv R package. The 95% CIs were calculated using the following formula: prediction ± 1.96 × standard error of prediction. We included antibody measurements only from 14 days before the first dose (setting the most recent measurement prior to 14 days before the first dose as 14 days) for those with no evidence of prior infection, and we excluded measurements taken after the 95th percentile of the observed *t* > 0 time points to avoid the outlier influence.

**Extended Data Fig. 10:**
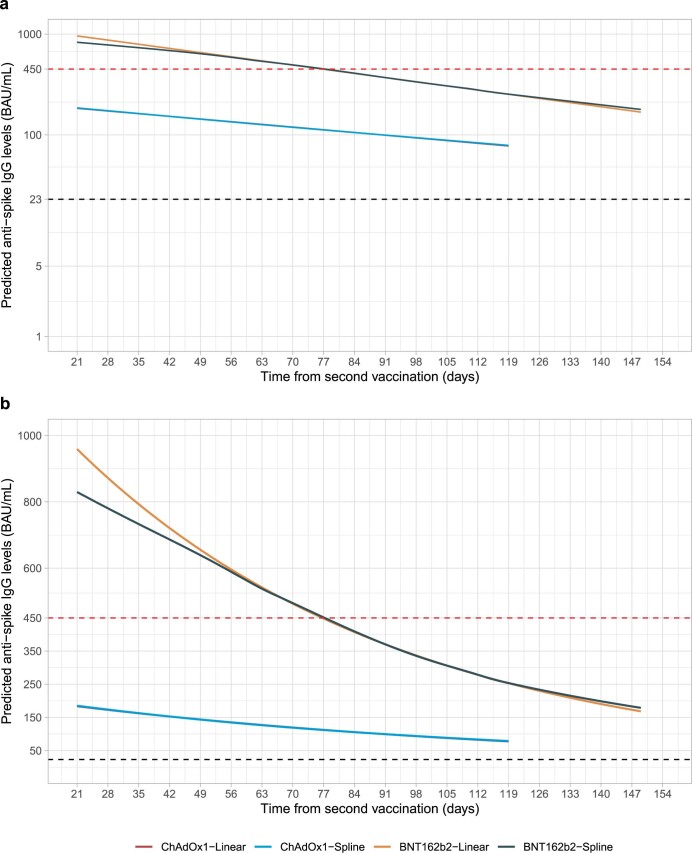
Comparison of linear exponential model with spline-based model in examining non-linearity of antibody decline. **a**, Plotted in log10 scale. **b**, Plotted in original scale. The estimated trajectory from the spline model (with 4 knots placed at 10^th^, 40^th^, 60^th^, and 90^th^ of observed time points) is similar with the linear exponential model for both ChAdOx1 and BNT162b2, indicating that there was no evidence of antibody decline flattening. Linear models provided better fit. Black dotted line shows the positivity threshold of 23 BAU/mL. Red dotted line shows the upper quantification limit of 450 BAU/mL. Source data

We used Bayesian linear mixed interval censored models to estimate changes in antibody levels after the second ChAdOx1 or BNT162b2 dose. We included measurements from 21 days after the second dose, reflecting the peak level from the GAMs (except for 3-week BNT162b2; see below). Measurements taken after the 95th percentile of the observed time points from 21 days after the second dose were excluded to avoid outlier influence. We assumed an exponential fall in antibody levels over time—that is, a linear decline on a log_2_ scale. To examine non-linearity in antibody declines, especially the assumption that the rate of antibody decline would flatten, we additionally fitted a model using four-knot splines for time (knots placed at 10th, 40th, 60th and 90th of observed time points) and compared the model fit with the log-linear model using the leave-one-out cross-validation information criterion (LOOIC). We found that the spline model had a higher LOOIC (indicating a worse model fit) than the log-linear model for ChAdOx1 (382,954 versus 378,640) and BNT162b2 (252,730 versus 240,472). For both vaccines, the estimated trajectories were similar, and there was no evidence of antibody decline flattening (Extended Data Fig. 10), so we used the log-linear model for the rest of the analysis.

Population-level fixed effects, individual-level random effects for intercept and slope and correlation among random effects were included in both models. The outcome was right-censored at 450 BAU ml^−1^, reflecting truncation of IgG values at the upper limit of quantification—that is, all measurements truncated to 450 BAU ml^−1^ were considered to be >450 BAU ml^−1^ in analyses. We built a multivariable model to examine the association between peak levels and antibody half-lives with continuous age (16–85 years), sex, ethnicity, report having a long-term health condition, report working in patient-facing healthcare, deprivation percentile, continuous dosing interval (7–13 weeks) and prior infection status for both vaccines. For BNT162b2, we additionally examined the effect of a 3-week dosing interval (17–28 days) by creating a binary variable and excluding antibody measurements ≤42 days after the second dose for the 3-week group (identified from the GAM as they peaked at around 42 days after the second dose).

For each Bayesian linear mixed interval censored model, weakly informative priors were used (Supplementary Table 9). Four chains were run per model with 4,000 iterations and a warm-up period of 2,000 iterations to ensure convergence, which was confirmed visually and by ensuring that the Gelman–Rubin statistic was less than 1.05 (Supplementary Table 10). 95% CrIs were calculated using highest posterior density intervals.

For the analysis of correlates of protection, we used data from study visits from 17 May 2021 to 4 October 2021. These visits were from 35–298 days after the first vaccination, which captured the time period over which real-world vaccine effectiveness has been observed to wane (Supplementary Fig. 4 and Supplementary Table 5). We grouped positive tests into episodes because PCR-positive results might be observed at multiple visits after infection. Following previous work, we defined the start of a new episode or ‘positive case’ as the date of (1) the first PCR-positive test in the study (not preceded by any study PCR-positive test); (2) a PCR-positive test after four or more consecutive negative visits; or (3) a PCR-positive test at least 120 days after the start of a previous episode with one or more negative tests immediately preceding this^12^. Analyses were based on visits, dropping any visits where participants were not at risk due to a recent new positive PCR test, with new PCR-positive episodes as the outcome. We used separate logistic GAMs for three outcomes: any positive PCR episode; a positive PCR episode with a moderate to high viral load (Ct value < 30); and a positive PCR episode with self-reported symptoms. Two exposure specifications were investigated. First, we considered the effect of the most recent antibody measurement obtained 21–59 days before the current visit, excluding more recent measurements to avoid changes in antibody levels arising from recent infection that might be detected only at the routine study visit despite occurring before this. Alternatively, we considered the maximum antibody measurement obtained ≥21 days before the visit. The relationship between antibody levels and the outcome was modeled using thin plate splines. We adjusted for the following confounders in all models: geographic area (12 regions in England or in Wales, Scotland or Northern Ireland) and age in years, rural/urban home address, sex, ethnicity (white versus non-white), household size, multi-generational household, deprivation, presence of long-term health conditions, working in a care home, having a patient-facing role in health or social care, direct or indirect contact with a hospital or care home, smoking status and visit frequency. Calendar time and age were included using a tensor spline, which was allowed to vary by region/country^12^.

Three groups were investigated: unvaccinated participants with or without evidence of prior infection (to assess the effect of prior infection); participants vaccinated with ChAdOx1; and participants vaccinated with BNT162b2. Vaccinated participants with evidence of prior infection were excluded, as there were insufficient data to model these groups separately, and the relationship between antibody levels and protection might differ in these groups. Visits occurring in the 21 days before vaccination were excluded, as we have previously reported that infection rates change in the run-up to vaccination^18^. Visits in vaccinated participants were included from ≥21 days after first vaccination. In secondary analyses, we grouped vaccinated individuals into one category.

Raw data were processed using Stata/MP 16. All analyses were performed in R version 3.6 using the following packages: tidyverse (version 1.3.0), mgcv (version 1.8–31), brms (version 2.14.0), rstanarm (version 2.21.1), splines (version 3.6.1), ggeffects (version 0.14.3), arsenal (version 3.4.0), cowplot (version 1.1.0) and bayesplot (version 1.7.2).

### Reporting Summary

Further information on research design is available in the Nature Research Reporting Summary linked to this article.

## Online content

Any methods, additional references, Nature Research reporting summaries, source data, extended data, supplementary information, acknowledgements, peer review information; details of author contributions and competing interests; and statements of data and code availability are available at 10.1038/s41591-022-01721-6.

## Supplementary information

Supplementary Information Supplementary Tables 1–10 and Supplementary Figs. 1–4

Reporting Summary

Supplementary Data 1 Source data for Supplementary Fig. 1

Supplementary Data 2 Source data for Supplementary Fig. 2

Supplementary Data 3 Source data for Supplementary Fig. 3

Supplementary Data 4 Source data for Supplementary Fig. 4

## Data Availability

Data are still being collected for the COVID-19 Infection Survey. De-identified study data are available for access by accredited researchers in the Office for National Statistics Secure Research Service (SRS) for accredited research purposes under Part 5, Chapter 5, of the Digital Economy Act 2017. Individuals can apply to be an accredited researcher using the short form on https://researchaccreditationservice.ons.gov.uk/ons/ONS_registration.ofml. Accreditation requires completion of a short free course on accessing the SRS. To request access to data in the SRS, researchers must submit a research project application for accreditation in the Research Accreditation Service. Research project applications are considered by the project team and the Research Accreditation Panel established by the UK Statistics Authority at regular meetings. Project application example guidance and an exemplar of a research project application are available. A complete record of accredited researchers and their projects is published on the UK Statistics Authority website to ensure transparency of access to research data. For further information about accreditation, contact Research.Support@ons.gov.uk or visit the SRS website. Source data are provided with this paper.

## Source data

Source Data Fig. 1 Statistical Source Data
Source Data Fig. 2 Statistical Source Data
Source Data Fig. 3 Statistical Source Data
Source Data Fig. 4 Statistical Source Data
Source Data Fig. 5 Statistical Source Data
Source Data Fig. 6 Statistical Source Data
Source Data Extended Data Fig. 2 Statistical Source Data
Source Data Extended Data Fig. 3 Statistical Source Data
Source Data Extended Data Fig. 4 Statistical Source Data
Source Data Extended Data Fig. 5 Statistical Source Data
Source Data Extended Data Fig. 6 Statistical Source Data
Source Data Extended Data Fig. 7 Statistical Source Data
Source Data Extended Data Fig. 8 Statistical Source Data
Source Data Extended Data Fig. 9 Statistical Source Data
Source Data Extended Data Fig. 10 Statistical Source Data

## Code availability

A copy of the analysis code is available at https://github.com/jiaweioxford/COVID19_second_vaccine_antibody_response.
