# Supplementary material for: Antibody responses and correlates of protection in the general population after two doses of the ChAdOx1 or BNT162b2 vaccines
Source: Nat Med. 2022 Feb 14;28(5):1072–82. doi: 10.1038/s41591-022-01721-6 (PMC9117148; doi:10.1038/s41591-022-01721-6)
Supplement: Supplementary file 1 — Supplementary Tables 1–10 and Supplementary Figs. 1–4 [file 41591_2022_1721_MOESM1_ESM.pdf]

nature portfolio

https://doi.org/10.1038/s41591-022-01721-6

Supplementary information

Antibody responses and correlates of protection in the general population after two doses of the ChAdOx1 or BNT162b2 vaccines

In the format provided by the authors and unedited

Supplementary information for

Antibody responses and correlates of protection in the general population after two doses of the ChAdOx1 or BNT162b2 vaccines

Jia Wei, Koen B. Pouwels, Nicole Stoesser, Philippa C. Matthews, Ian Diamond, Ruth Studley, Emma Rourke, Duncan Cook, John I Bell, John N Newton, Jeremy Farrar, Alison Howarth, Brian D. Marsden, Sarah Hoosdally, E Yvonne Jones, David I Stuart, Derrick W. Crook, Tim E. A. Peto, A. Sarah Walker, David W. Eyre and the COVID-19 Infection Survey team

Contents

- **Supplementary Tables 1-10**
- **Supplementary Figures 1-4**

Supplementary Tables

	ChAdOx1 two dose no prior infection (N=121,322)	BNT162b2 two dose no prior infection (N=79,693)	ChAdOx1 two dose prior infection (N=12,066)	BNT162b2 two dose prior infection (N=9,412)	Total (N=222,493)	p value
**Percentage (%)**	54.5	35.8	5.4	4.2	100	
**Dosing interval (days)**						
**Median**	76	71	76	65	74	
**Q1, Q3**	68, 78	58, 77	66, 78	56, 76	64, 78	
**Age (years)**						< 0.001
**Median**	58	56	53	37	57	
**Q1, Q3**	48, 67	36, 70	45, 62	29, 57	43, 68	
**Sex**						< 0.001
**Female**	64,470 (53.1%)	44,717 (56.1%)	6,477 (53.7%)	5,202 (55.3%)	120,866 (54.3%)	
**Male**	56,852 (46.9%)	34,976 (43.9%)	5,589 (46.3%)	4,210 (44.7%)	101,627 (45.7%)	
**Ethnicity**						< 0.001
**Non-white**	5,698 (4.7%)	4,858 (6.1%)	932 (7.7%)	1,107 (11.8%)	12,595 (5.7%)	
**White**	115,624 (95.3%)	74,835 (93.9%)	11,134 (92.3%)	8,305 (88.2%)	209,898 (94.3%)	
**Household size**						< 0.001
**1**	22,809 (18.8%)	15,069 (18.9%)	1,928 (16.0%)	1,291 (13.7%)	41,097 (18.5%)	
**2**	59,704 (49.2%)	39,965 (50.1%)	5,065 (42.0%)	3,890 (41.3%)	108,624 (48.8%)	
**3**	18,176 (15.0%)	11,691 (14.7%)	2,167 (18.0%)	1,859 (19.8%)	33,893 (15.2%)	
**4**	15,215 (12.5%)	9,143 (11.5%)	2,075 (17.2%)	1,593 (16.9%)	28,026 (12.6%)	
**5+**	5,418 (4.5%)	3,825 (4.8%)	831 (6.9%)	779 (8.3%)	10,853 (4.9%)	
**Deprivation percentile**						< 0.001
**Median**	64	62	60	56	62	
**Q1, Q3**	40, 83	38, 82	35, 81	32, 78	39, 82	
**Report working in patient facing healthcare**						< 0.001
**No**	119,937 (98.9%)	74,840 (93.9%)	11,805 (97.8%)	8,840 (93.9%)	215,422 (96.8%)	
**Yes**	1,385 (1.1%)	4,853 (6.1%)	261 (2.2%)	572 (6.1%)	7,071 (3.2%)	
**Report having a long-term health condition**						< 0.001
**No**	87,034 (71.7%)	56,147 (70.5%)	9,083 (75.3%)	7,415 (78.8%)	159,679 (71.8%)	
**Yes**	34,288 (28.3%)	23,546 (29.5%)	2,983 (24.7%)	1,997 (21.2%)	62,814 (28.2%)	

Supplementary Table 1. Characteristics of participants with two vaccine doses and at least one antibody measurement from 91 days before the first vaccination through to 4th October. Continuous variables were compared using Kruskall-Wallis tests, and categorical variables were compared using one-sided Chi-squared tests.

(a)						
			Without prior infection		With prior infection	
**Time from second dose (days)**	**Dosing interval (days)**	**Age (years)**	**ChAdOx1**	**BNT162b2**	**ChAdOx1**	**BNT162b2**
**21**	21	60	NA	276 (236-324)	NA	305 (212-440)
**21**	56	60	159 (147-172)	371 (340-405)	312 (219-443)	418 (284-614)
**21**	70	60	175 (168-182)	400 (386-415)	325 (278-380)	425 (355-509)
**21**	84	60	169 (161-178)	392 (367-418)	280 (218-360)	426 (308-588)
**42**	21	60	NA	328 (284-378)	NA	347 (243-495)
**42**	56	60	132 (123-141)	357 (324-394)	277 (197-390)	392 (261-587)
**42**	70	60	137 (132-142)	375 (359-391)	294 (251-344)	406 (342-481)
**42**	84	60	143 (137-150)	356 (330-383)	274 (215-347)	395 (286-547)
**63**	21	60	NA	287 (249-331)	NA	371 (260-530)
**63**	56	60	109 (101-116)	321 (295-350)	230 (163-326)	372 (241-572)
**63**	70	60	111 (107-115)	321 (310-333)	254 (217-298)	379 (318-453)
**63**	84	60	121 (116-127)	323 (304-344)	266 (209-339)	395 (288-542)

(b)						
			Without prior infection		With prior infection	
**Time from second dose (days)**	**Dosing interval (days)**	**Age (years)**	**ChAdOx1**	**BNT162b2**	**ChAdOx1**	**BNT162b2**
**-49**	70	20	108 (90-130)	295 (208-417)	351 (317-388)	347 (273-440)
**21**	70	20	183 (154-217)	317 (225-447)	404 (362-450)	420 (308-574)
**-49**	70	40	81 (76-87)	247 (203-300)	219 (208-231)	293 (243-353)
**21**	70	40	156 (148-165)	289 (241-348)	394 (377-413)	410 (342-492)
**-49**	70	60	65 (62-68)	255 (216-301)	142 (135-150)	234 (195-282)
**21**	70	60	175 (168-182)	325 (278-380)	400 (386-415)	425 (355-509)
**-49**	70	80	37 (33-42)	112 (85-148)	91 (83-100)	141 (107-185)
**21**	70	80	175 (160-193)	254 (193-334)	404 (377-432)	422 (324-548)

Supplementary Table 2. Predicted anti-spike IgG levels (BAU/mL) with 95% confidence interval (CI) from generalised additive models. a, Comparison of 8-, 10-, 12-, and 3-week dosing interval 21, 42, 63 days after the second dose in 60-year-olds. **b,** Comparison of 20-, 40-, 60-, and 80-year-olds 21 days after the first and second dose in those with 10-week dosing interval. The 95% confidence intervals are calculated by prediction ± 1.96*standard error of prediction. Values truncated at 450 BAU/mL counted as =450 BAU/mL.

(a) Frequency of antibody measurements per participant		
	**ChAdOx1**	**BNT162b2**
1	37,478	17,863
2	39,268	16,444
3	20,450	13,839
4	3,442	6,074
5	1	832
6	0	1
**(b) Frequency of antibody measurements relative to the second vaccination**		
(days)	**ChAdOx1**	**BNT162b2**
21-28	15,693	6,964
28-35	16,403	7,161
35-42	16,535	6,934
42-49	16,427	7,061
49-56	16,200	6,973
56-63	16,788	7,310
63-70	16,284	7,507
70-77	15,750	7,471
77-84	14,258	7,709
84-91	12,962	7,925
91-98	11,070	8,064
98-105	8,873	7,934
105-112	7,219	7,215
112-119	5,886	6,436
119-126	789	5,837
126-133	0	4,721
133-140	0	3,732
140-147	0	2,831
147-149	0	945

Supplementary Table 3. Frequency of antibody measurements per participant and frequency of antibody measurements relative to the time of the second vaccination in the Bayesian linear mixed model. 100,639 ChAdOx1 participants contributed 191,137 antibody measurements ≥21 days after the second dose, median (IQR) [range] 2 (1-2) [1-5] measurements per participant. 55,053 BNT162b2 participants contributed 120,728 antibody measurements ≥21 days after the second dose, median (IQR) [range] 2 (1-3) [1-6] per participant. The antibody measurements were taken a median (IQR) [range] 61 (41-83) [21-119] days after the second vaccination for ChAdOx1 and 79 (51-106) [21-149] days for BNT162b2.

		ChAdOx1			BNT162b2		
		Posterior mean	95% Crl		Posterior mean	95% Crl	
**Unadjusted baseline**	Peak level (Intercept)	184	183	185	959	944	974
	IgG half-life (slope)	79	78	80	51	50	52
**Adjusted baseline**	Peak level (Intercept)	160	155	162	976	903	1011
	IgG half-life (slope)	81	79	83	52	50	53
**Age**	Peak level: 60 years						
	IgG half-life: 60 years						
	Change in peak level: per 10-years older	**3**	**2**	**4**	**-76**	**-84**	**-68**
	Change in half-life: per 10-years older	**-1**	**-1**	**0**	0	0	0
**Sex**	Peak level: Female						
	IgG half-life: Female						
	Change in peak level: Male	**-5**	**-7**	**-3**	**-140**	**-164**	**-117**
	Change in half-life: Male	0	-1	2	-1	-2	1
**Ethnicity**	Peak level: White						
	IgG half-life: White						
	Change in peak level: Non-white	**41**	**36**	**47**	**141**	**78**	**208**
	Change in half-life: Non-white	**-8**	**-11**	**-5**	0	-3	2
**Report having a long-term health condition**	Peak level: No						
	IgG half-life: No						
	Change in peak level: Yes	**-5**	**-7**	**-3**	**-79**	**-104**	**-55**
	Change in half-life: Yes	**-2**	**-4**	**-1**	**-1**	**-2**	**0**
**Report working in patient-facing healthcare**	Peak level: No						
	IgG half-life: No						
	Change in peak level: Yes	**10**	**2**	**19**	**287**	**221**	**358**
	Change in half-life: Yes	0	-6	6	**-5**	**-6**	**-3**
**Deprivation**	Peak level: 60 (median)						
	IgG half-life: 60 (median)						
	Change in peak level: per 10 percentile higher	**-1**	**-1**	**-1**	**5**	**1**	**10**
	Change in half-life: per 10 percentile higher	**1**	**0**	**1**	0	0	0
**3-week interval**	Peak level: No						
	IgG half-life: No						

	Change in ‘peak level’: Yes (vs 8 weeks) *				**-163**	**-227**	**-95**
	Change in half-life: Yes (vs 8 weeks)				**6**	**2**	**10**
**Dosing interval**	Peak level: 8 weeks (median)						
	IgG half-life: 8 weeks (median)						
	Change in peak level: per 1 week longer	**6**	**5**	**7**	**12**	**1**	**23**
	Change in half-life: per 1 week longer	0	-1	0	0	0	1
**Prior infection**	Peak level: No						
	IgG half-life: No						
	Change in peak level: Yes	**219**	**210**	**227**	**312**	**248**	**381**
	Change in half-life: Yes	**13**	**9**	**17**	**11**	**8**	**15**

Supplementary Table 4. Posterior mean and 95% credible intervals for anti-spike IgG peak level (intercept) (BAU/mL) and half-life (slope) (days) in the unadjusted models and multivariable models in 100,639 participants with two ChAdOx1 doses and 55,053 participants with two BNT162b2 doses. The reference categories in the multivariable model are: 60-year-old, female, white ethnicity, not reporting a long-term health condition, not a healthcare worker, deprivation percentile=60, 8-week dosing interval, and no prior infection. Bold numbers indicate an effect not compatible with chance (95% credible interval excludes no effect). *The predicted peak level in 3-week vs 8-week is extrapolated and for modelling purpose only, it should not be interpreted as an actual peak.

Days from first vaccination	ChAdOx1	BNT162b2
**35-49**	1,364	1,875
**49-63**	4,766	4,327
**63-77**	9,681	5,027
**77-91**	14,081	5,830
**91-105**	18,268	7,318
**105-119**	22,644	9,288
**119-133**	26,749	10,725
**133-147**	27,934	11,090
**147-161**	28,251	12,327
**161-175**	27,636	13,392
**175-189**	26,770	14,111
**189-203**	21,210	14,195
**203-217**	14,786	13,397
**217-231**	10,285	11,202
**231-245**	4,640	7,638
**245-259**	993	3,966
**259-273**	65	1,673
**273-287**	1	647
**287-298**	0	103

Supplementary Table 5. Frequency of visits relative to the time of the first vaccination in the generalised additive models estimating correlates of protection. Study visits were included from 17th May 2021 to 4th October 2021, i.e. while the Delta variant accounted for nearly all cases. The median (IQR) [range] was 149 (115-182) [36-273] days for ChAdOx1 and 167 (123-204) [35-298] days for BNT162b2. Note that UK started its booster programme from 16th September 2021, so a small number of visits occurred in the first two weeks after initiating the booster campaign.

a)		ChAdOx1			BNT162b2		
		Total	Non-responder	%	Total	Non-responder	%
First dose	At least one antibody measurement >=21 days after first dose and before second dose	57,650	4,280	7.4	30,843	1,374	4.5
	Two or more antibody measurements, including at least one antibody measurement >=21 days after first dose and before second dose	29,277	1,706	5.8	15,525	546	3.5
Second dose	At least one antibody measurement >=21 days after second dose	100,776	1,010	1	57,313	312	0.5
	Two or more antibody measurements, including at least one antibody measurement >=21 days after second dose	77,947	496	0.6	44,311	146	0.3
**b)**		**ChAdOx1**			**BNT162b2**		
First and second dose	At least one Ab measurement >=21 days after first dose and before second dose, and after second dose	**Second dose: non-responder**	**Second dose: responder**	**Total**	**Second dose: non-responder**	**Second dose: responder**	**Total**
	**First dose: non-responder**	192 (0.5%)	3,243 (7.2%)	3,435 (7.7%)	37 (0.2%)	1,036 (5.8%)	1,073 (6.0%)
	**First dose: responder**	99 (0.2%)	41,212 (92.1%)	41,311 (92.3%)	15 (0.1%)	16,909 (93.9%)	16,924 (94.0%)
	**Total**	291 (0.7%)	44,455 (99.3%)	44,746 (100%)	52 (0.3%)	17,945 (99.7%)	17,997 (100%)
	Two or more antibody measurements, including at least one antibody measurement >=21 days after first dose and before second dose, and after second dose	**Second dose: non-responder**	**Second dose: responder**	**Total**	**Second dose: non-responder**	**Second dose: responder**	**Total**
	**First dose: non-responder**	59 (0.3%)	1,092 (5.7%)	1,151 (6.0%)	8 (0.1%)	364 (5.1%)	372 (5.2%)
	**First dose: responder**	10 (0.1%)	17,965 (93.9%)	17,975 (94.0%)	0 (0%)	6,731 (94.8%)	6,731 (94.8%)
	**Total**	69 (0.4%)	19,057 (99.6%)	19,126 (100%)	8 (0.1%)	7,095 (99.9%)	7,103 (100%)

Supplementary Table 6. Percentages of non-responders to first or second dose of ChAdOx1 or BNT162b2 using a heuristic rule as all antibody measurements being <16 BAU/mL and having at least one antibody measurement 21 days after the first or second dose. a, Non-responders in those who had antibody measurements after first dose or second dose. **b,** Non-responders in those who had antibodies measured after both first and second dose by responses to each dose. To examine the robustness of this definition, this rule was further applied in those having at least two antibody measurements.

	Responders (N=215,746)	Non-responders (N=6,747)	Total (N=222,493)	p value
**Dosing interval (days)**				< 0.001
**Median**	74	77	74	
**Q1, Q3**	64, 78	70, 79	64, 78	
**Age (years)**				< 0.001
**Median**	56	63	57	
**Q1, Q3**	43, 67	53, 72	43, 68	
**Sex**				< 0.001
**Female**	11,7859 (54.6%)	3,007 (44.6%)	120,866 (54.3%)	
**Male**	97,887 (45.4%)	3,740 (55.4%)	101,627 (45.7%)	
**Ethnicity**				< 0.001
**Non-white**	12,383 (5.7%)	212 (3.1%)	12,595 (5.7%)	
**White**	203,363 (94.3%)	6,535 (96.9%)	209,898 (94.3%)	
**Household size**				< 0.001
**1**	39,556 (18.3%)	1,541 (22.8%)	41,097 (18.5%)	
**2**	105,134 (48.7%)	3,490 (51.7%)	108,624 (48.8%)	
**3**	33,046 (15.3%)	847 (12.6%)	33,893 (15.2%)	
**4**	27,405 (12.7%)	621 (9.2%)	28,026 (12.6%)	
**5+**	10,605 (4.9%)	248 (3.7%)	10,853 (4.9%)	
**Deprivation percentile**				< 0.001
**Median**	63	60	62	
**Q1, Q3**	39, 82	36, 81	39, 82	
**Report working in patient facing healthcare**				< 0.001
**No**	208,765 (96.8%)	6,657 (98.7%)	215,422 (96.8%)	
**Yes**	6,981 (3.2%)	90 (1.3%)	7,071 (3.2%)	
**Report having a long-term health condition**				< 0.001
**No**	156,009 (72.3%)	3,670 (54.4%)	159,679 (71.8%)	
**Yes**	59,737 (27.7%)	3,077 (45.6%)	62,814 (28.2%)	

Supplementary Table 7. Comparison of the characteristics of responders with non-responders to first or second dose of ChAdOx1 or BNT162b2. Non-responders were identified using a heuristic rule as all antibody measurements being <15 BAU/mL and having at least one antibody measurement 21 days after the first or second dose. Continuous variables were compared using Kruskall-Wallis tests, and categorical variables were compared using one-sided Chi-squared tests.

Number of antibody measurements	0 (N=179,855)	1-2 (N=92,397)	3-4 (N=78,565)	≥5 (N=51,531)	Total (N=402,348)
**Dosing interval (days)**					
Median	74	74	74	75	74
Q1, Q3	62, 78	63, 78	64, 77	67, 78	63, 78
**Age (years)**					
Median	55	54	56	60	56
Q1, Q3	39, 69	41, 67	43, 67	49, 69	41, 68
**Sex**					
Female	95,435 (53.1%)	49,544 (53.6%)	42,821 (54.5%)	28,501 (55.3%)	216,301 (53.8%)
Male	84,420 (46.9%)	42,853 (46.4%)	35,744 (45.5%)	23,030 (44.7%)	186,047 (46.2%)
**Ethnicity**					
Non-white	13,874 (7.7%)	5,099 (5.5%)	4,521 (5.8%)	2,974 (5.8%)	26,468 (6.6%)
White	165,981 (92.3%)	87,298 (94.5%)	74,044 (94.2%)	48,557 (94.2%)	375,880 (93.4%)
**Household size**					
1	36,725 (20.4%)	15,928 (17.2%)	14,239 (18.1%)	10,930 (21.2%)	77,822 (19.3%)
2	82,100 (45.6%)	43,323 (46.9%)	38,341 (48.8%)	26,960 (52.3%)	190,724 (47.4%)
3	27,952 (15.5%)	14,580 (15.8%)	12,301 (15.7%)	7,012 (13.6%)	61,845 (15.4%)
4	22,978 (12.8%)	12,970 (14.0%)	10,027 (12.8%)	5,029 (9.8%)	51,004 (12.7%)
5+	10,100 (5.6%)	5,596 (6.1%)	3,657 (4.7%)	1,600 (3.1%)	20,953 (5.2%)
**Deprivation percentile**					
Median	61	63	62	63	62
Q1, Q3	37, 81	40, 82	38, 82	39, 83	38, 82
**Report working in patient facing healthcare**					
No	175,361 (97.5%)	89,467 (96.8%)	76,008 (96.7%)	49,947 (96.9%)	390,783 (97.1%)
Yes	4,494 (2.5%)	2,930 (3.2%)	2,557 (3.3%)	1,584 (3.1%)	11,565 (2.9%)
**Report having a long-term health condition**					
No	130,595 (72.6%)	67,246 (72.8%)	56,574 (72.0%)	35,859 (69.6%)	290,274 (72.1%)
Yes	49,260 (27.4%)	25,151 (27.2%)	21,991 (28.0%)	15,672 (30.4%)	112,074 (27.9%)

Supplementary Table 8. Comparison of the characteristics of all participants ≥16 years in the COVID-19 Infection Survey who received two ChAdOx1 or two BNT162b2 vaccinations from 8th December 2020 to 4th October 2021 by number of antibody measurements. 179,855 participants did not provide blood samples for antibody testing. 222,493 participants provided blood samples (92,397 had 1-2 measurements, 78,565 had 3-4 measurements, and 51,531 had ≥5 measurements) and were included in the analyses.

Model term	Priors for ChAdOx1	Priors for BNT162b2
**Intercept**	normal (7.5,1)	normal (9.5, 1)
**Slope**	normal (0, 0.1)	normal (0, 0.1)
**Coefficient for change in intercept (age)**	normal (0, 1)	normal (0, 1)
**Coefficient for change in slope (age)**	normal (0, 0.1)	normal (0, 0.1)
**Coefficient for change in intercept (sex)**	normal (0, 1)	normal (0, 1)
**Coefficient for change in slope (sex)**	normal (0, 0.1)	normal (0, 0.1)
**Coefficient for change in intercept (ethnicity)**	normal (0, 1)	normal (0, 1)
**Coefficient for change in slope (ethnicity)**	normal (0, 0.1)	normal (0, 0.1)
**Coefficient for change in intercept (long-term health condition)**	normal (0, 1)	normal (0, 1)
**Coefficient for change in slope (long-term health condition)**	normal (0, 0.1)	normal (0, 0.1)
**Coefficient for change in intercept (healthcare worker)**	normal (0, 1)	normal (0, 1)
**Coefficient for change in slope (healthcare worker)**	normal (0, 0.1)	normal (0, 0.1)
**Coefficient for change in intercept (deprivation)**	normal (0, 1)	normal (0, 1)
**Coefficient for change in slope (deprivation)**	normal (0, 0.01)	normal (0, 0.01)
**Coefficient for change in intercept (3-week interval)**	normal (0, 1)	normal (0, 1)
**Coefficient for change in slope (3-week interval)**	normal (0, 0.1)	normal (0, 0.1)
**Coefficient for change in intercept (dosing interval)**	normal (0, 1)	normal (0, 1)
**Coefficient for change in slope (dosing interval)**	normal (0, 0.1)	normal (0, 0.1)
**Coefficient for change in intercept (prior infection)**	normal (0, 1)	normal (0, 1)
**Coefficient for change in slope (prior infection)**	normal (0, 0.1)	normal (0, 0.1)
**Random effect SD: intercept**	cauchy(0, 0.5)	cauchy(0, 0.5)
**Random effect SD: slope**	cauchy(0, 0.01)	cauchy(0, 0.01)
**Random effect intercept & slope covariance**	lkj_corr_cholesky(2)	lkj_corr_cholesky(2)

Supplementary Table 9. Priors used in the Bayesian linear mixed interval-censored models. Age (16-85 years) and deprivation percentile (0-100) were scaled by dividing by 10; dosing interval (42-91 days) was scaled by dividing by 7.

		Estimate	Est.Error	95%CrI		Rhat	Bulk_ESS	Tail_ESS
**ChAdOx1**	Intercept	7.3174	0.0111	7.2951	7.3391	1.0023	1375	2882
	time	-0.0124	0.0002	-0.0127	-0.0121	1.0000	6611	5322
	age	0.0254	0.0034	0.0187	0.0321	1.0024	1593	3235
	sex	-0.0466	0.0083	-0.0631	-0.0306	1.0015	1399	2808
	ethnicity	0.3331	0.0202	0.2946	0.3737	1.0040	1501	2532
	lthc	-0.0450	0.0093	-0.0632	-0.0267	1.0049	1102	2022
	hcw	0.0903	0.0374	0.0164	0.1634	1.0017	1509	2453
	deprivation	-0.0103	0.0016	-0.0134	-0.0072	1.0018	1550	2878
	dosing interval	0.0524	0.0036	0.0454	0.0593	1.0020	1560	2920
	prior infection	1.2453	0.0158	1.2146	1.2765	1.0038	1746	3266
	time:age	-0.0001	0.0000	-0.0002	0.0000	1.0014	6671	6023
	time:sex	0.0001	0.0001	-0.0001	0.0003	1.0006	6612	6153
	time:ethnicity	-0.0013	0.0003	-0.0019	-0.0008	1.0004	5712	5471
	time:lthc	-0.0004	0.0001	-0.0006	-0.0001	1.0005	6501	5556
	time:hcw	-0.0001	0.0005	-0.0010	0.0008	1.0005	6041	4866
	time:deprivation	0.0001	0.0000	0.0001	0.0001	1.0007	7110	5923
	time:dosing interval	-0.0001	0.0001	-0.0002	0.0000	1.0002	6541	5819
	time:prior infection	0.0017	0.0002	0.0012	0.0021	1.0000	6010	5228
	sigma	0.4757	0.0014	0.4729	0.4785	1.0131	400	677
	sd(Intercept)	1.0101	0.0028	1.0045	1.0157	1.0100	1090	2678
	sd(time)	0.0017	0.0002	0.0013	0.0022	1.0170	319	387
	cor(Intercept,time)	-0.2679	0.0159	-0.2983	-0.2367	1.0001	3372	4253
**BNT162b2**	Intercept	9.9300	0.0259	9.8796	9.9810	1.0003	3553	4861
	time	-0.0194	0.0003	-0.0200	-0.0188	1.0004	6401	6408
	age	-0.1167	0.0065	-0.1297	-0.1040	1.0003	4746	6190
	sex	-0.2235	0.0187	-0.2600	-0.1868	1.0007	4743	6293
	ethnicity	0.1944	0.0430	0.1106	0.2788	1.0010	4377	5850
	lthc	-0.1222	0.0196	-0.1601	-0.0843	1.0006	4213	5716
	hcw	0.3717	0.0397	0.2961	0.4521	1.0019	4062	4894
	deprivation	0.0075	0.0034	0.0007	0.0143	1.0006	4495	6470
	3-week	-0.2638	0.0571	-0.3754	-0.1489	1.0002	3648	5632
	dosing interval	0.0183	0.0085	0.0017	0.0351	1.0002	3599	5597
	prior infection	0.4002	0.0377	0.3270	0.4747	1.0000	4529	5485
	time:age	0.0000	0.0001	-0.0001	0.0002	1.0003	7850	6895
	time:sex	-0.0002	0.0002	-0.0006	0.0002	1.0003	8799	6686
	time:ethnicity	-0.0002	0.0005	-0.0012	0.0008	1.0007	7573	6634
	time:lthc	-0.0005	0.0002	-0.0009	0.0000	1.0009	8150	6849
	time:hcw	-0.0019	0.0004	-0.0027	-0.0011	1.0012	6397	6317
	time:deprivation	0.0000	0.0000	-0.0001	0.0001	1.0013	9273	6197
	time:3-week	0.0019	0.0006	0.0007	0.0031	0.9999	5061	6104
	time:dosing interval	0.0001	0.0001	-0.0001	0.0003	1.0010	7100	6346
	time:prior infection	0.0035	0.0005	0.0025	0.0044	0.9998	7001	6688
	sigma	0.8319	0.0036	0.8248	0.8389	1.0027	1982	4821
	sd(Intercept)	1.1414	0.0122	1.1175	1.1655	1.0032	2434	4901
	sd(time)	0.0027	0.0002	0.0023	0.0032	1.0180	315	396
	cor(Intercept,time)	-0.9189	0.0447	-0.9882	-0.8234	1.0203	283	324

Supplementary Table 10. Model coefficients and MCMC diagnostics for the multivariable Bayesian linear mixed models.

Supplementary Figures

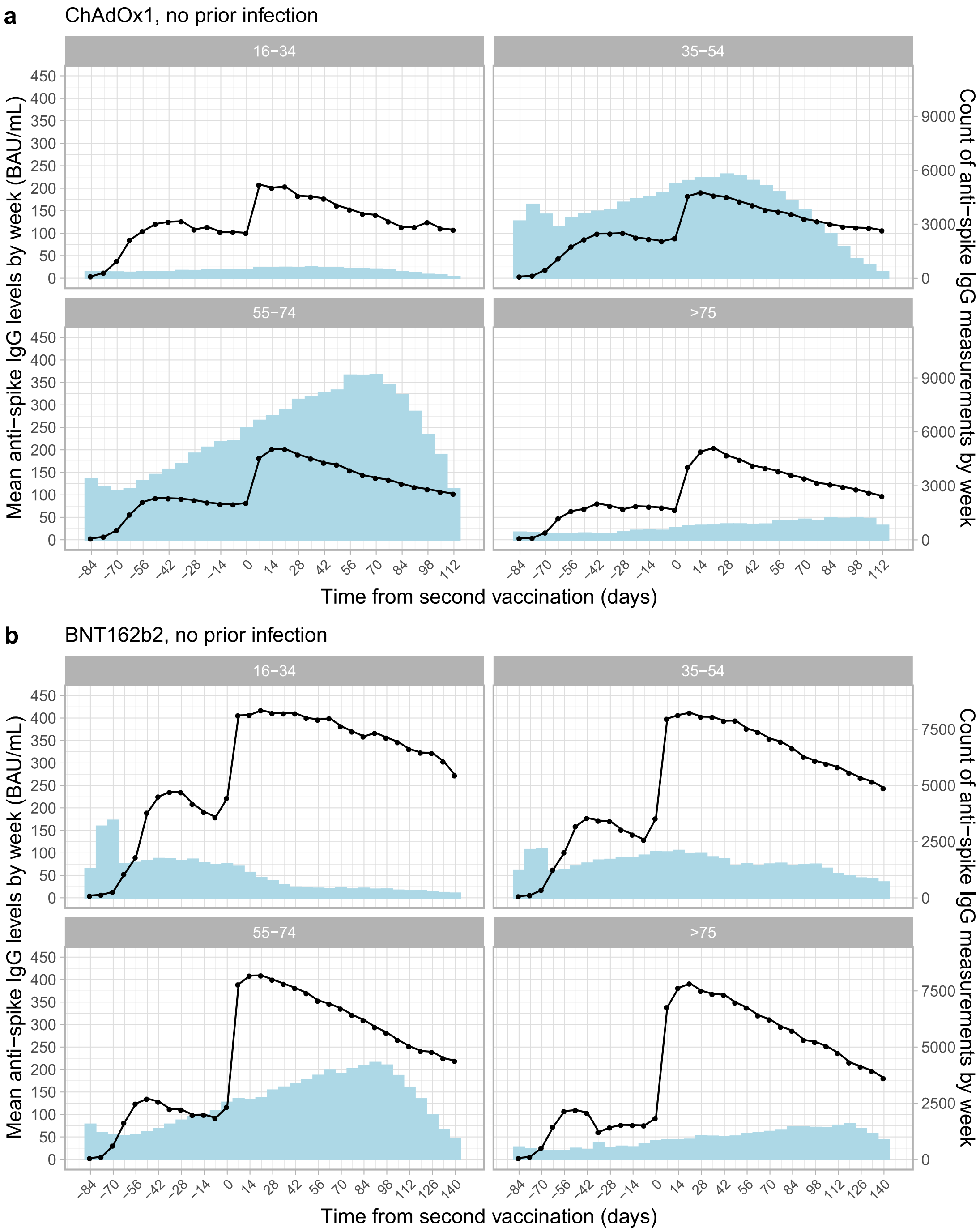

Supplementary Fig. 1. Unadjusted mean anti-spike IgG levels (BAU/mL) and count of IgG measurements by week from second vaccination in those without prior infection. Plots are separated by age group (16-34, 35-54, 55-74, and ≥75 years) and are not separated by dosing intervals. **a**, in those who received two ChAdOx1 vaccinations. **b**, in those who received two BNT162b2 vaccinations. Values truncated at 450 BAU/mL counted as =450 BAU/mL.

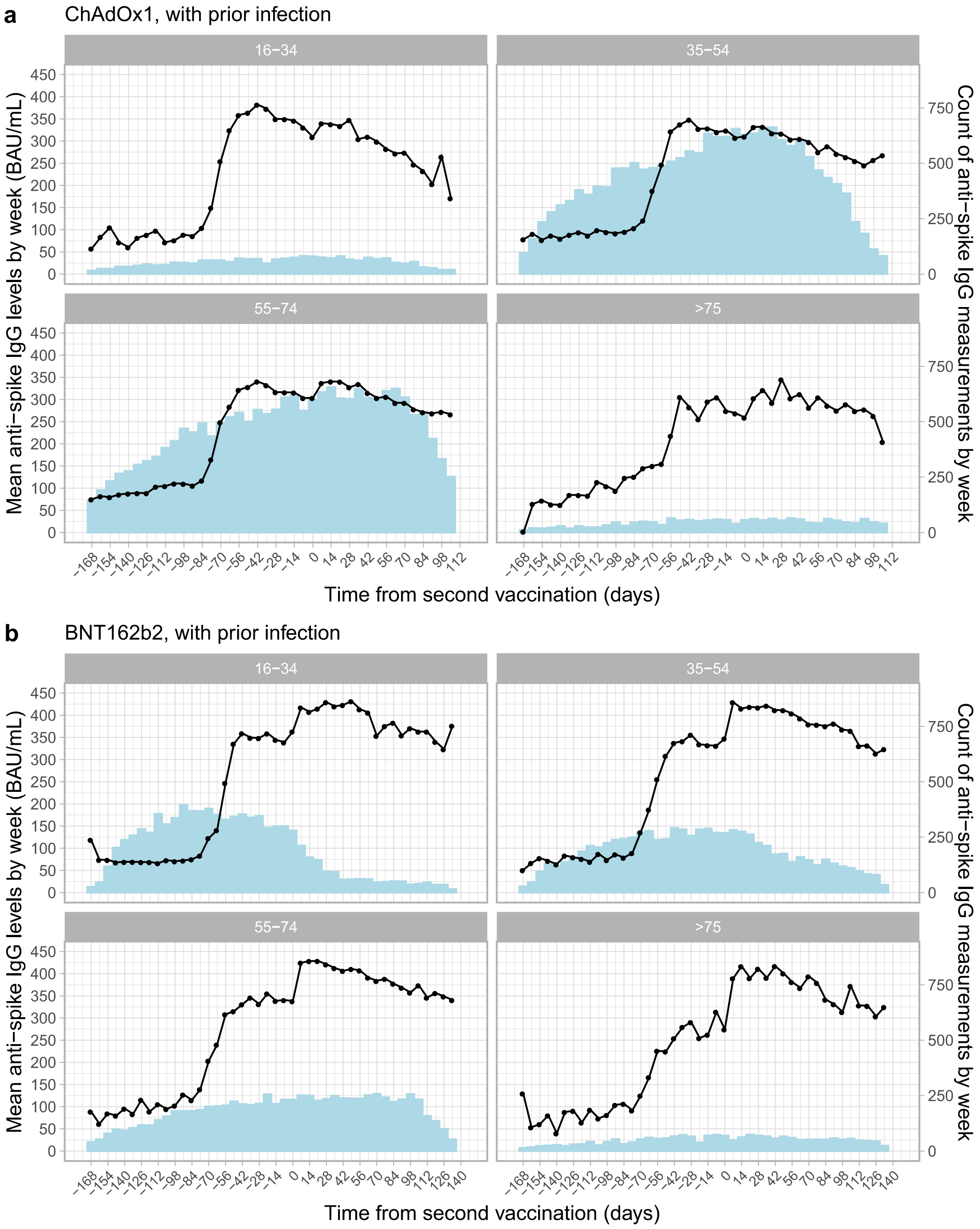

Supplementary Fig. 2. Unadjusted mean anti-spike IgG levels (BAU/mL) and count of IgG measurements by week from second vaccination in those with prior infection. Plots are separated by age group (16-34, 35-54, 55-74, and ≥75 years) and are not separated by dosing intervals. **a**, in those who received two ChAdOx1 vaccinations. **b**, in those who received two BNT162b2 vaccinations. Values truncated at 450 BAU/mL counted as =450 BAU/mL.

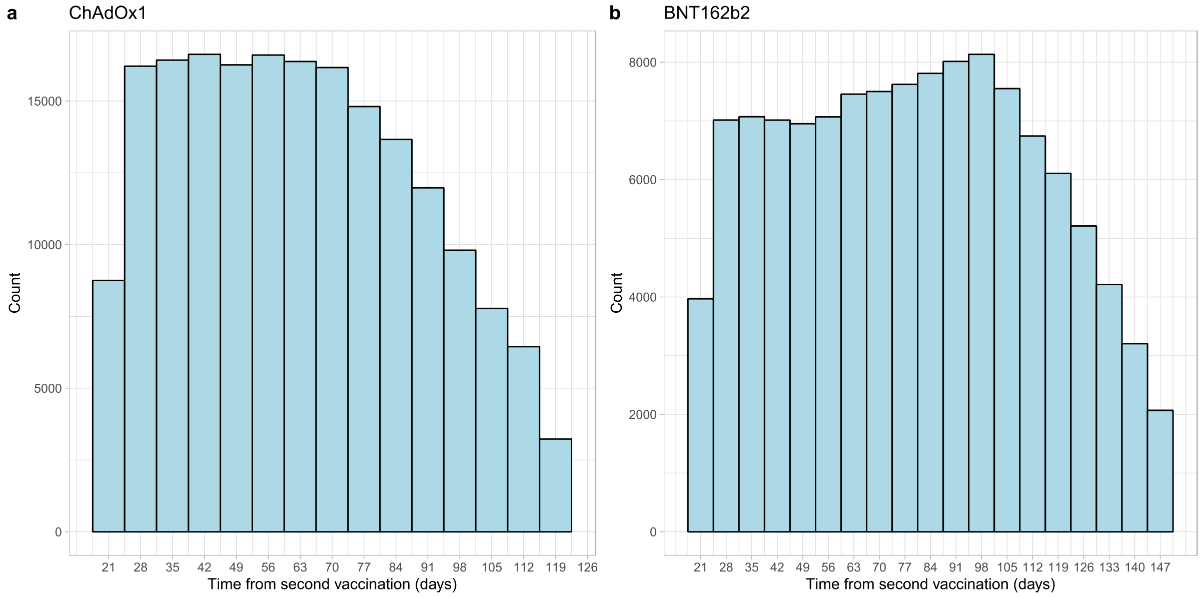

Supplementary Fig. 3. Distribution of antibody measurements relative to the time of the second vaccination in the Bayesian linear mixed model. The median (IQR) [range] was 61 (41-83) [21-119] days for ChAdOx1 and 79 (51-106) [21-149] days for BNT162b2.

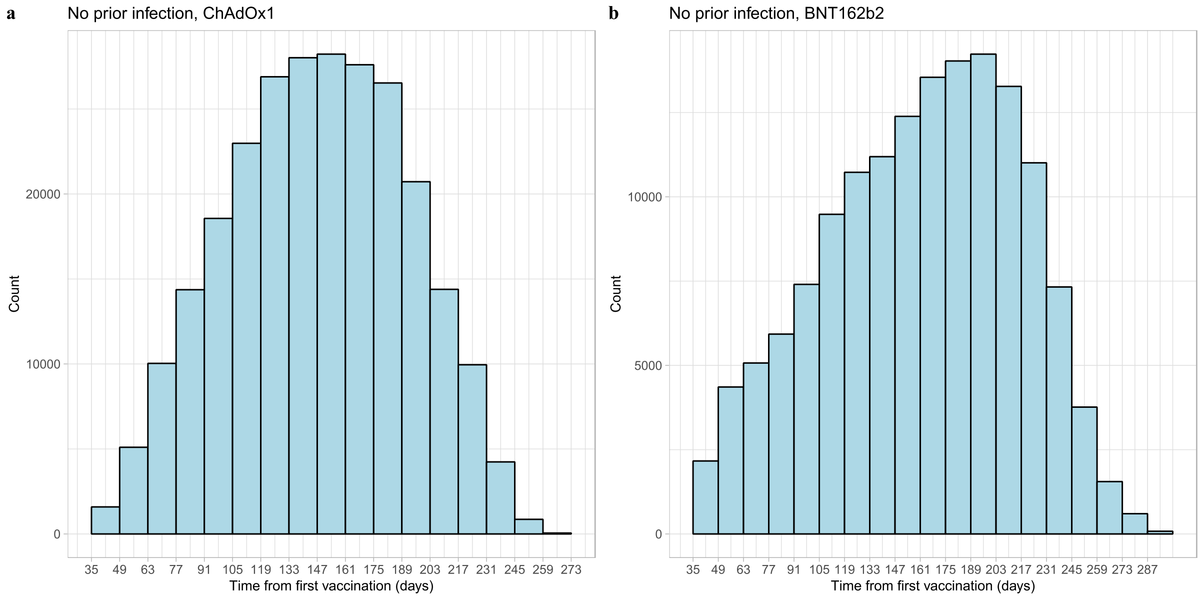

Supplementary Fig. 4. Distribution of study visits relative to the time of the first vaccination in the generalised additive models estimating correlates of protection. Study visits were included from 17th May 2021 to 4th October 2021, i.e. while the Delta variant accounted for nearly all cases. The median (IQR) [range] was 149 (115-182) [36-273] days for ChAdOx1 and 167 (123-204) [35-298] days for BNT162b2.
